# The Effects of Concurrent Aerobic and Strength Training on Muscle Fiber Hypertrophy: A Systematic Review and Meta-Analysis

**DOI:** 10.1007/s40279-022-01688-x

**Published:** 2022-04-27

**Authors:** Tommy R. Lundberg, Joshua F. Feuerbacher, Marvin Sünkeler, Moritz Schumann

**Affiliations:** 1 Division of Clinical Physiology, Department of Laboratory Medicine, Karolinska Institute, Stockholm, Sweden; 2 Unit of Clinical Physiology, Karolinska University Hospital, Stockholm, Sweden; 3 Institute of Cardiovascular Research and Sports Medicine, Department of Molecular and Cellular Sports Medicine, German Sport University, Am Sportpark Müngersdorf 6, 50933 Cologne, Germany

## Abstract

**Background:**

Whole muscle hypertrophy does not appear to be negatively affected by concurrent aerobic and strength training compared to strength training alone. However, there are contradictions in the literature regarding the effects of concurrent training on hypertrophy at the myofiber level.

**Objective:**

The current study aimed to systematically examine the extent to which concurrent aerobic and strength training, compared with strength training alone, influences type I and type II muscle fiber size adaptations. We also conducted subgroup analyses to examine the effects of the type of aerobic training, training modality, exercise order, training frequency, age, and training status.

**Design:**

A systematic literature search was conducted according to the Preferred Reporting Items for Systematic Reviews and Meta-Analyses (PRISMA) [PROSPERO: CRD42020203777]. The registered protocol was modified to include only muscle fiber hypertrophy as an outcome.

**Data Sources:**

PubMed/MEDLINE, ISI Web of Science, Embase, CINAHL, SPORTDiscus, and Scopus were systematically searched on 12 August, 2020, and updated on 15 March, 2021.

**Eligibility Criteria:**

Population: healthy adults of any sex and age; intervention: supervised, concurrent aerobic and strength training of at least 4 weeks; comparison: identical strength training prescription, with no aerobic training; and outcome: muscle fiber hypertrophy.

**Results:**

A total of 15 studies were included. The estimated standardized mean difference based on the random-effects model was − 0.23 (95% confidence interval [CI] − 0.46 to − 0.00, *p* = 0.050) for overall muscle fiber hypertrophy. The standardized mean differences were − 0.34 (95% CI − 0.72 to 0.04, *p* = 0.078) and − 0.13 (95% CI − 0.39 to 0.12, *p* = 0.315) for type I and type II fiber hypertrophy, respectively. A negative effect of concurrent training was observed for type I fibers when aerobic training was performed by running but not cycling (standardized mean difference − 0.81, 95% CI − 1.26 to − 0.36). None of the other subgroup analyses (i.e., based on concurrent training frequency, training status, training modality, and training order of same-session training) revealed any differences between groups.

**Conclusions:**

In contrast to previous findings on whole muscle hypertrophy, the present results suggest that concurrent aerobic and strength training may have a small negative effect on fiber hypertrophy compared with strength training alone. Preliminary evidence suggests that this interference effect may be more pronounced when aerobic training is performed by running compared with cycling, at least for type I fibers.

**Supplementary Information:**

The online version contains supplementary material available at 10.1007/s40279-022-01688-x.

## Key Points

**Table:** 

In this meta-analysis, we report that concurrent aerobic and strength training can attenuate muscle fiber hypertrophy compared with strength training alone.
This interference effect is relatively small and may be more pronounced when aerobic training is performed by running compared with cycling, at least for type I fibers.
None of the other subgroup analyses (concurrent training frequency, training status, training modality, and training order of same-session training) revealed any differences between concurrent training and strength training alone.

## Introduction

Concurrent training refers to the combination of aerobic and strength training to simultaneously develop aerobic capacity and muscle strength and/or hypertrophy. Current physical activity guidelines recommend that all children, adolescents, and adults engage in concurrent training to promote significant health benefits [1]. Concurrent training is also typically recommended for individuals who need effective countermeasures against physical deconditioning, e.g., because of aging, disease, or injury. Apart from the health perspective, many sports require athletes to simultaneously incorporate divergent training modalities into their training regimen. Because the typical adaptations to aerobic and strength training alone represent opposite ends of the adaptation continuum, the question has been raised as to whether skeletal muscle can comply with concurrent aerobic and strength training stimuli, without compromising the desired adaptations [2, 3].

To address this issue, we recently conducted an updated systematic review and meta-analysis examining the effects of concurrent aerobic and strength training on gains in muscle mass, maximal strength, and explosive strength [4]. The results showed that although the increase in explosive strength may be attenuated by concurrent aerobic and strength training, whole-muscle hypertrophy, muscle mass, and maximal strength development do not seem to be compromised. However, a thorough examination of the individual studies we reviewed for our meta-analysis revealed that there are great inconsistencies in the literature regarding the effects of concurrent training on muscle hypertrophy. More specifically, none of the individual studies that reported an interference effect on muscle hypertrophy used the most reliable techniques to assess muscle size (i.e., magnetic resonance imaging [MRI] or computed tomography [CT]). Instead, all except one study [5] used fiber size as the outcome measure for muscle hypertrophy. This raises the question of whether fiber hypertrophy might be affected differently compared to changes in whole muscle size following concurrent training. In support, de Souza et al. reported that increases in fiber size were attenuated with concurrent training, whereas measurements at the whole muscle level showed no interference effect [6].

There are several possible reasons for these contradictory findings. At the muscle fiber level, it is generally agreed that strength training, but not aerobic training, augments myofibrillar protein accretion [7]. Thus, long-term resistance training increases muscle fiber size, whereas long-term aerobic training induces morphological and metabolic changes in skeletal muscle that result in improved endurance and fatigue resistance, but have comparatively little effect on fiber size [8]. Acute studies attempting to find mechanistic explanations for an interference effect on muscle hypertrophy have shown conflicting results [9–11]. Although Babcock et al. reported that concurrent aerobic and strength exercise attenuated the satellite cell response compared with strength exercise alone [12], subsequent studies failed to demonstrate that the anabolic effect induced by aerobic-type exercise was antagonistic to the molecular response induced by strength exercise [13, 14]. Considering that assessment of myofibrillar protein content and the measurement of fiber size are associated with large inter-biopsy variations, and that the concurrent training studies reporting them generally suffer from small sample sizes, it is possible that some of these contradictory results may be explained by methodological shortcomings.

Aside from the notion that fiber hypertrophy after concurrent training may be affected differently than changes in whole muscle size, previous studies suggest that the effect of concurrent training may also be modulated by training design variables such as training modality, volume/frequency, and training status [13–15]. Therefore, it is reasonable to also examine the effects of plausible moderators of fiber type-specific hypertrophy during concurrent training. To overcome the above limitations and knowledge gaps, the current study aimed to systematically examine the extent to which concurrent aerobic and strength training, compared with strength training alone, influences type I and type II muscle fiber size adaptations. We also conducted subgroup analyses to examine the effects of the type of aerobic training, training modality, exercise order, training frequency, age, and training status.

## Methods

### Systematic Literature Search

This study reports a subgroup analysis of a previously registered systematic review and meta-analysis (PROSPERO: CRD42020203777). For the purposes of this study, the registered protocol was modified to include only muscle fiber hypertrophy as an outcome. Therefore, some of the methods presented here have been described previously [4].

A systematic literature search was conducted according to the Preferred Reporting Items for Systematic Reviews and Meta-Analyses (PRISMA). The PubMed/MEDLINE, ISI Web of Science, Embase, CINAHL, SPORTDiscus, and Scopus databases were systematically searched using a search string specifically adapted to the search requirements for each database (Table S1 of the Electronic Supplementary Material [ESM]).

**Fig. 1:**
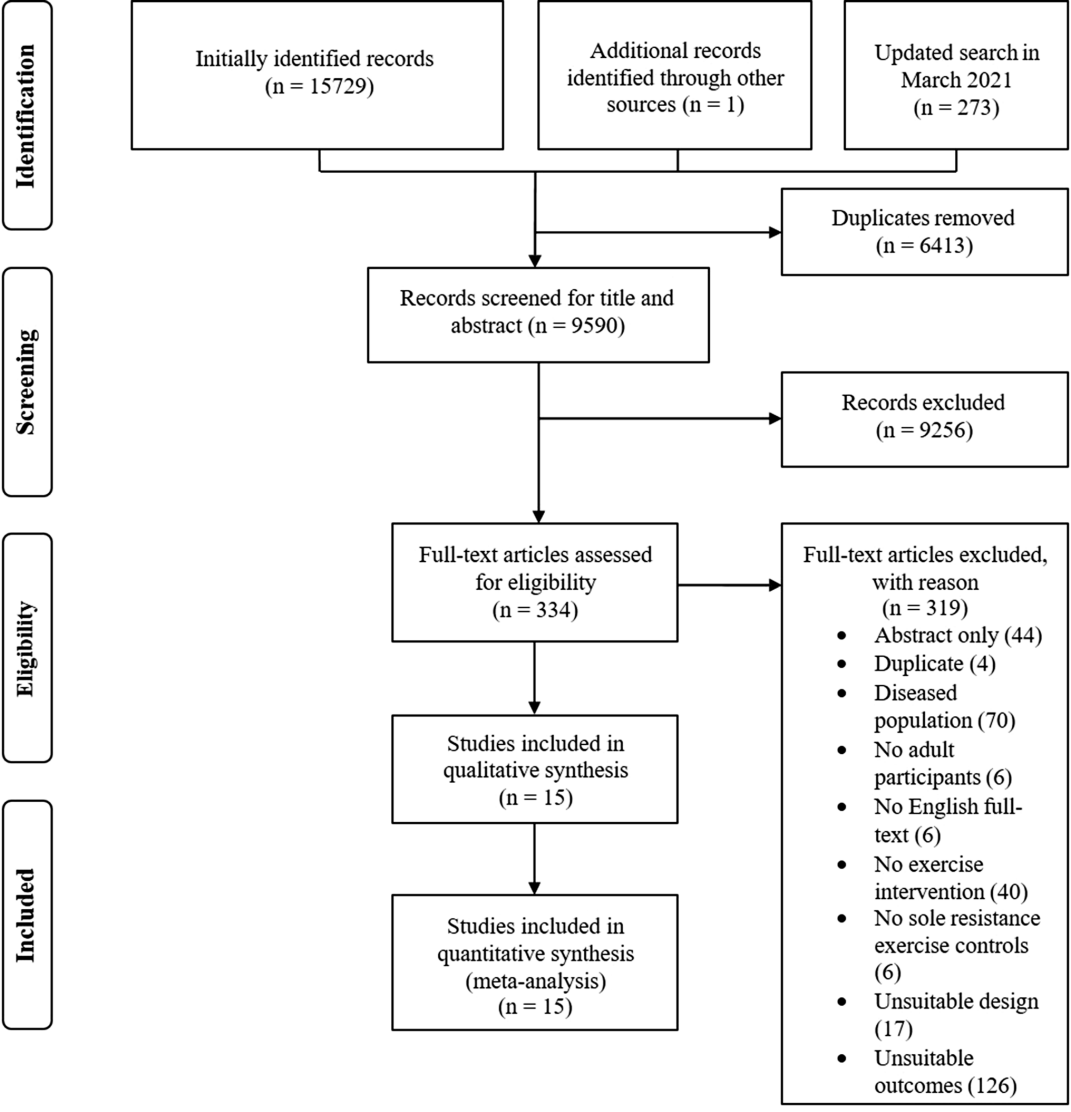
Flowchart of the search process and study selection

The search was conducted on 12 August, 2020, and updated on 15 March, 2021. The literature search was performed independently by two researchers and included saving the online search, removing duplicates, and screening titles, abstracts, and full texts. Potential conflicts were resolved by consulting with a third author. In addition, a gray literature search was performed by screening Google Scholar and the reference lists of previously identified eligible full texts. A flowchart of the search process and study selection is shown in Fig. 1.

### Eligibility Criteria

Inclusion criteria were defined based on the Population, Intervention, Control and Outcomes (PICO) criteria [16]. The population included healthy adults with no sex or age restrictions. The intervention had to consist of supervised combined aerobic and strength training of at least 4 weeks. For a comparison, eligible studies had to include a group performing strength training alone with an identical strength training prescription. Outcomes of interest were defined as hypertrophy of type I and type II fibers. Exclusion criteria included languages other than English and German, abstracts and dissertations, cross-sectional studies assessing only acute exercise responses, and observational studies.

### Data Extraction

Data extraction was performed independently by two authors. The following data were extracted from each included study: (1) the general characteristics (e.g., author(s), year of publication, and aim of the study); (2) participant information (e.g., sample size, training status, and age); (3) intervention data for all groups (e.g., intervention duration, type of interventions); and (4) specific outcomes (changes in both type I and II muscle fiber hypertrophy). If the mean and standard deviation of each group were not provided, authors of the primary studies were contacted to request the data at baseline and post-intervention. If data were presented in a graph and no additional data were provided upon request, mean and standard deviation were extracted using WebPlotDigitizer version 4.4 (Pacifica, CA, USA) [17].

### Data Synthesis and Analyses

Standardized mean differences (SMD) for each group were calculated using the mean difference divided by the pre-test standard deviation for each group separately, whereby the difference between these effects is considered the SMD. An inverse variance-weighted random-effects model was fitted to the effect sizes and 95% confidence intervals (CIs) were calculated around the mean. This model was selected because variance was expected owing to the heterogeneity in study designs. Additionally, log variability ratios were calculated. Meta-analyses were performed using R (3.6.2), RStudio (1.2.5033), and the metafor package (version 2.4.0) [18]. Effect sizes were calculated for *pre-test post-test control group designs* using raw score standardization as previously recommended [19, 20] and the exact sampling variance of effect sizes was calculated as recommended [19].

Heterogeneity (i.e., *τ*^2^), was estimated using the restricted maximum-likelihood estimator [21]. To complete the heterogeneity analyses, the *Q* test for heterogeneity [22] and the *I*^2^ statistic [23] were also calculated, with values of 20%, 50%, and 75% indicating low, moderate, and high heterogeneity, respectively [24]. Studentized residuals and Cook’s distances were examined to assess whether studies might be outliers and/or overly influential [25]. A trim-and-fill funnel plot was created to estimate the number of studies that might be missing from the meta-analysis (Fig. S1 of the ESM). The rank correlation test [26] and regression test [27] using the standard error of the observed outcomes as the predictor were used to examine the SMDs for funnel plot asymmetry.

Effect sizes from studies with more than two intervention or control groups were combined in accordance with the recommendations of the Cochrane Handbook by recalculating the mean and pooling the standard deviation of each intervention group [28], except for a subgroup analysis where different interventions from individual studies were included in separate subgroups. Similarly, pre-data and post-data from studies that assessed variances of type II fibers (e.g., type IIa and IIb/IIx fibers) were combined for an interindividual comparison.

Subgroup analyses were conducted for aerobic training type (i.e., cycling vs running), concurrent training frequency (i.e., low frequency of 4.5 ± 0.8 vs high frequency of 6.3 ± 0.8 weekly sessions, equating to 2.2 ± 0.3 vs 3.2 ± 0.4 weekly sessions in the strength training-only group), training status (i.e. untrained vs active), and training modality (i.e. concurrent training on different days vs concurrent training on the same day vs concurrent training in the same session). The thresholds for training frequency were determined with the aim of comparing two balanced groups. Because of inconsistent reporting in the original articles, no further distinctions in terms of training status were made. For studies comparing concurrent training in the same session, we also compared the training order (i.e., aerobic training before strength training vs strength training before aerobic training) if a sufficient number of studies was available. Studies were placed into subgroups based on the description provided in the manuscript. This was particularly true for training status: studies were classified as “untrained” if participants were clearly described as “sedentary,” “previously untrained,” or “inactive”. Conversely, all other studies were classified as “active” (i.e., “recreationally active,” “trained,” and “well-trained”).

### Assessment of Methodological Quality

Risk of bias for the included studies was assessed using the Physiotherapy Evidence Database (PEDro) scale independently by two authors. The PEDro scale has previously been assessed as a valid measure of the methodological quality of randomized trials [29]. Studies with scores > 6 were considered to be of “high quality,” studies with scores of 4–5 were considered to be of “medium quality,” and studies with scores < 4 were considered to be of “low quality”. The following sources of bias were considered: selection (sequence generation and allocation concealment), performance (blinding of participants/personnel), detection (blinding outcome assessors), attrition (incomplete outcome data), reporting (selective reporting), and other potential bias (e.g., recall bias). The risk of bias scores for the included studies are shown in Table S2 of the ESM. The mean score for criteria 2–11 of the PEDro-scale was 4.3 ± 0.8, indicating a medium quality.

## Results

### Study Characteristics

The final analysis included 15 studies that examined both type I and II fiber hypertrophy. A total of 300 participants were included, of which 153 participants performed supervised combined aerobic and strength training and 147 participants performed strength training alone. Among the included studies, cycling was the most common type of aerobic training (12 studies), followed by running (three studies).

### Overall Muscle Fiber Hypertrophy

**Fig. 2:**
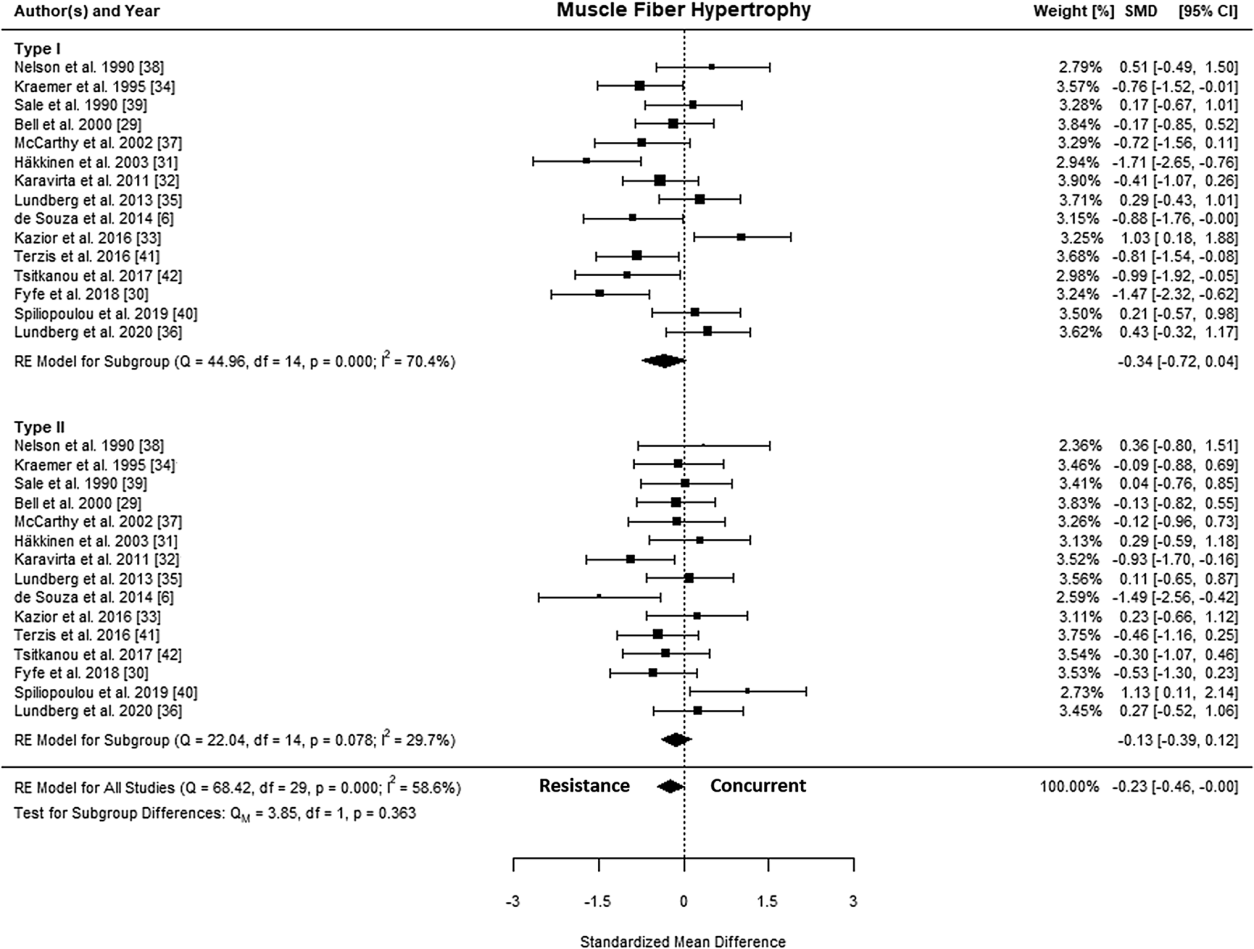
Forest plot comparing differences in hypertrophy of type I and type II fibers. *CI* confidence interval, *SMD* standardized mean difference, *RE* random effects model

A total of 15 studies [6, 30–43] were included in the quantitative analysis. The SMDs ranged from − 1.71 to 1.13, and the estimated SMD based on the random-effects model was − 0.23 (95% CI − 0.46 to − 0.00, *p* = 0.050). The forest plot showing the observed outcomes and the estimate based on the random-effects model is shown in Fig. 2. The *Q* test revealed that the true outcomes appear to be heterogeneous (*Q*(29) = 68.42, *p* < 0.001, *τ*^2^ = 0.24, *I*^2^ = 58.6%). The estimated average log variability ratio based on the random-effects model was − 0.12 (95% CI − 0.26 to 0.02, *p* = 0.097).

### Type I Muscle Fiber Hypertrophy

**Fig. 3:**
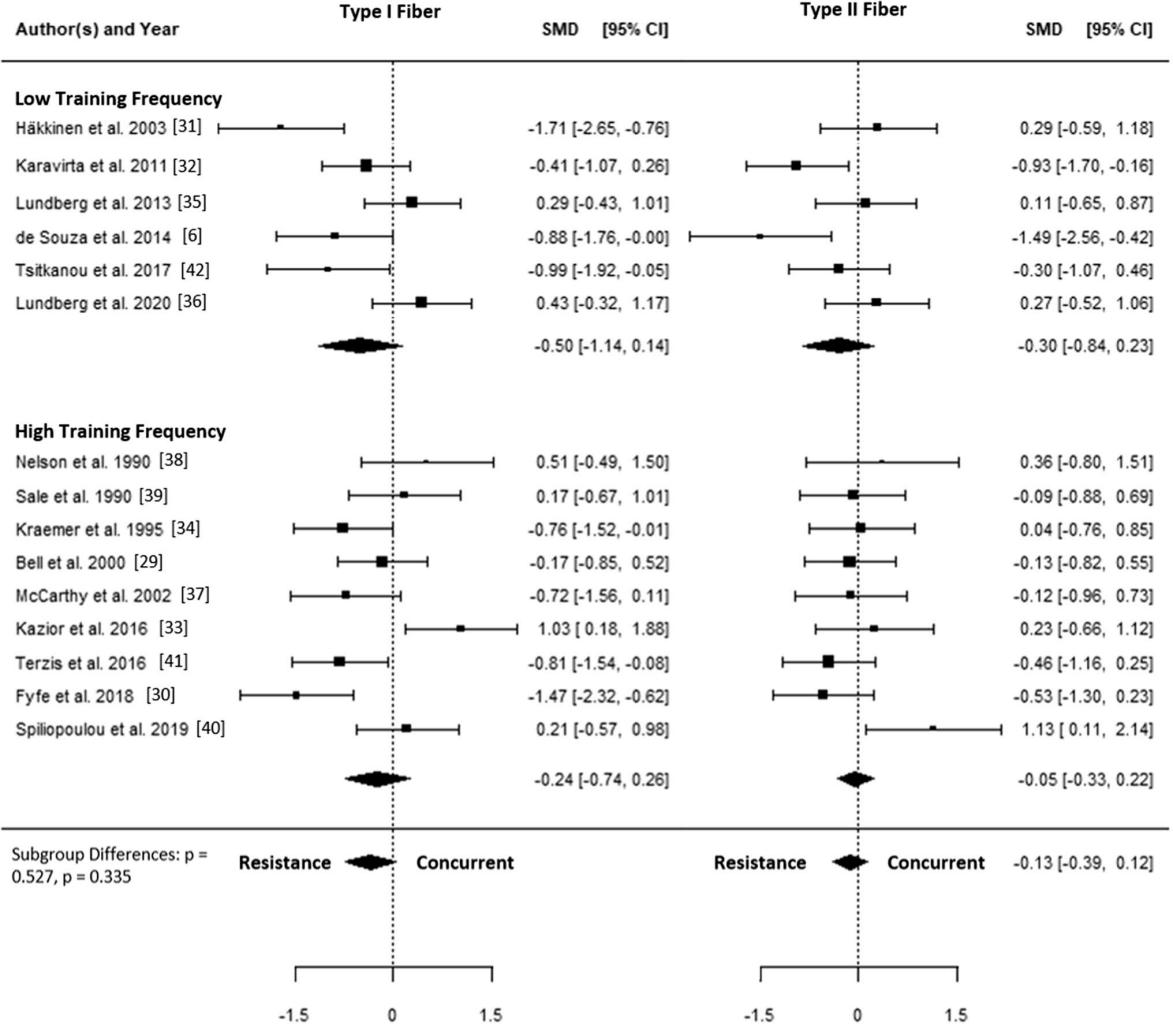
Forest plot comparing differences in hypertrophy of type I and II fibers between low and high training frequency. *CI* confidence interval, *SMD* standardized mean difference

**Fig. 4:**
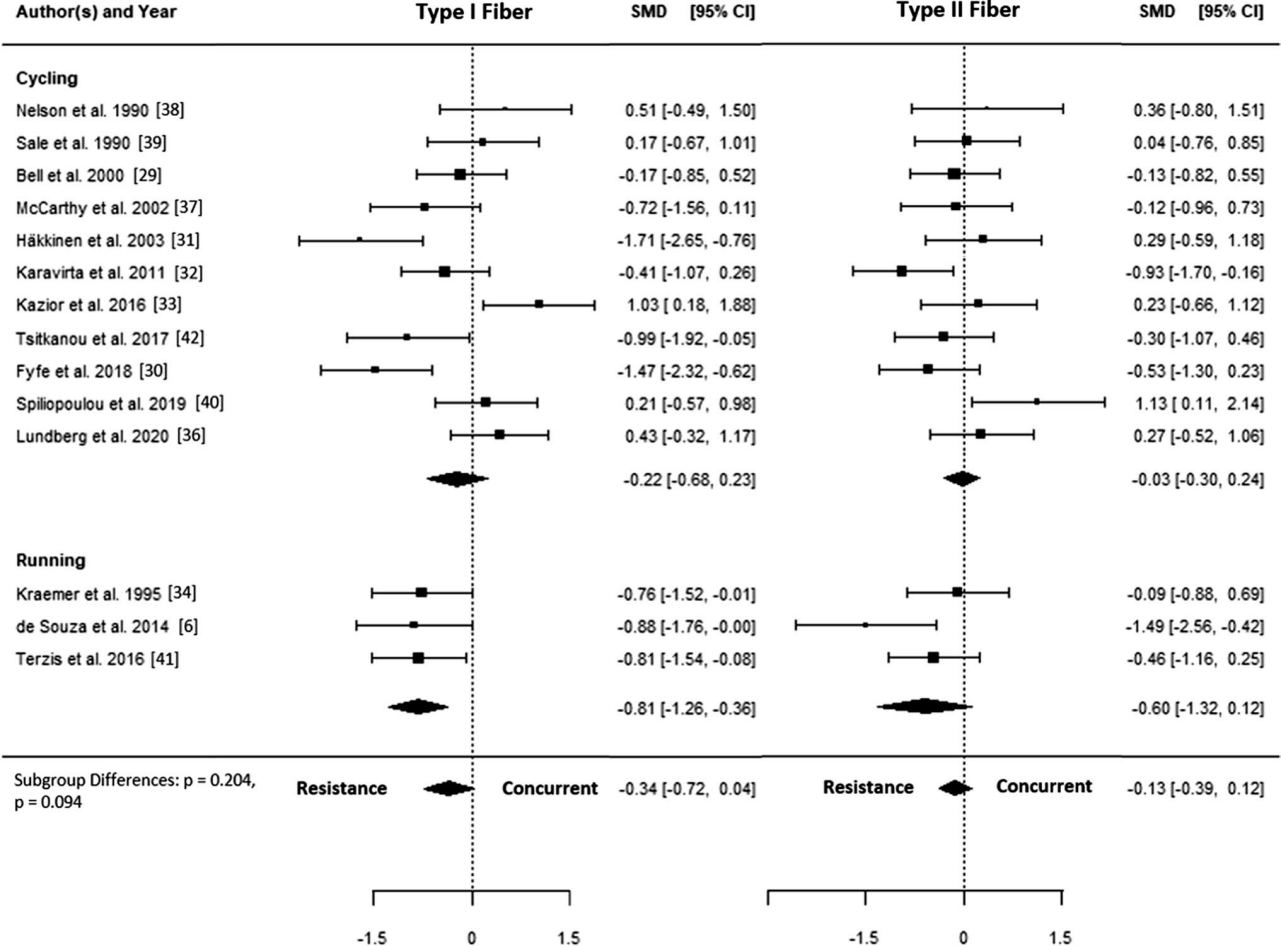
Forest plot comparing differences in hypertrophy of type I and II fibers separated by type of aerobic training. *CI* confidence interval, *SMD* standardized mean difference

**Fig. 5:**
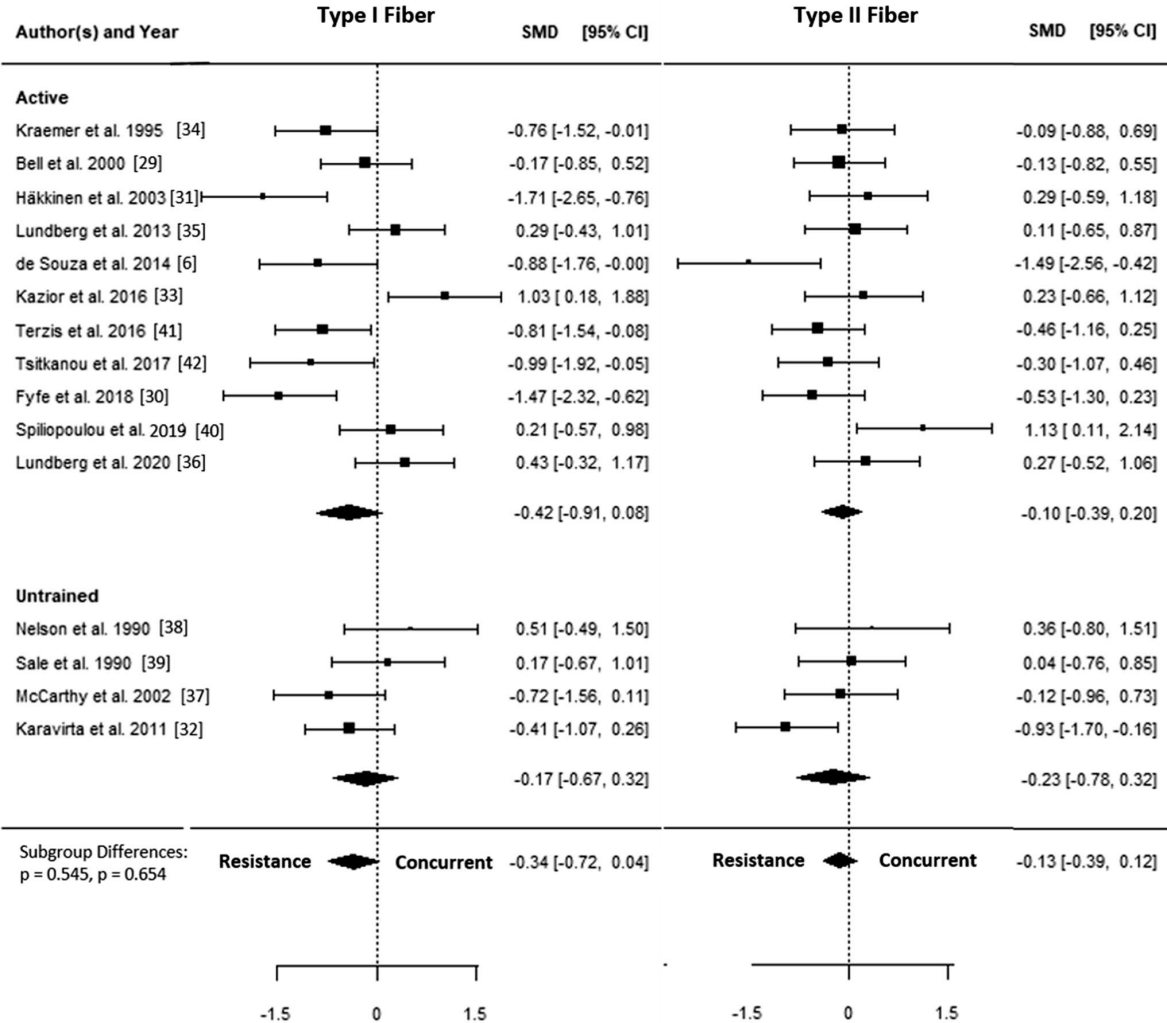
Forest plot comparing differences in hypertrophy of type I and II fibers between active and untrained participants. *CI* confidence interval, *SMD* standardized mean difference

**Fig. 6:**
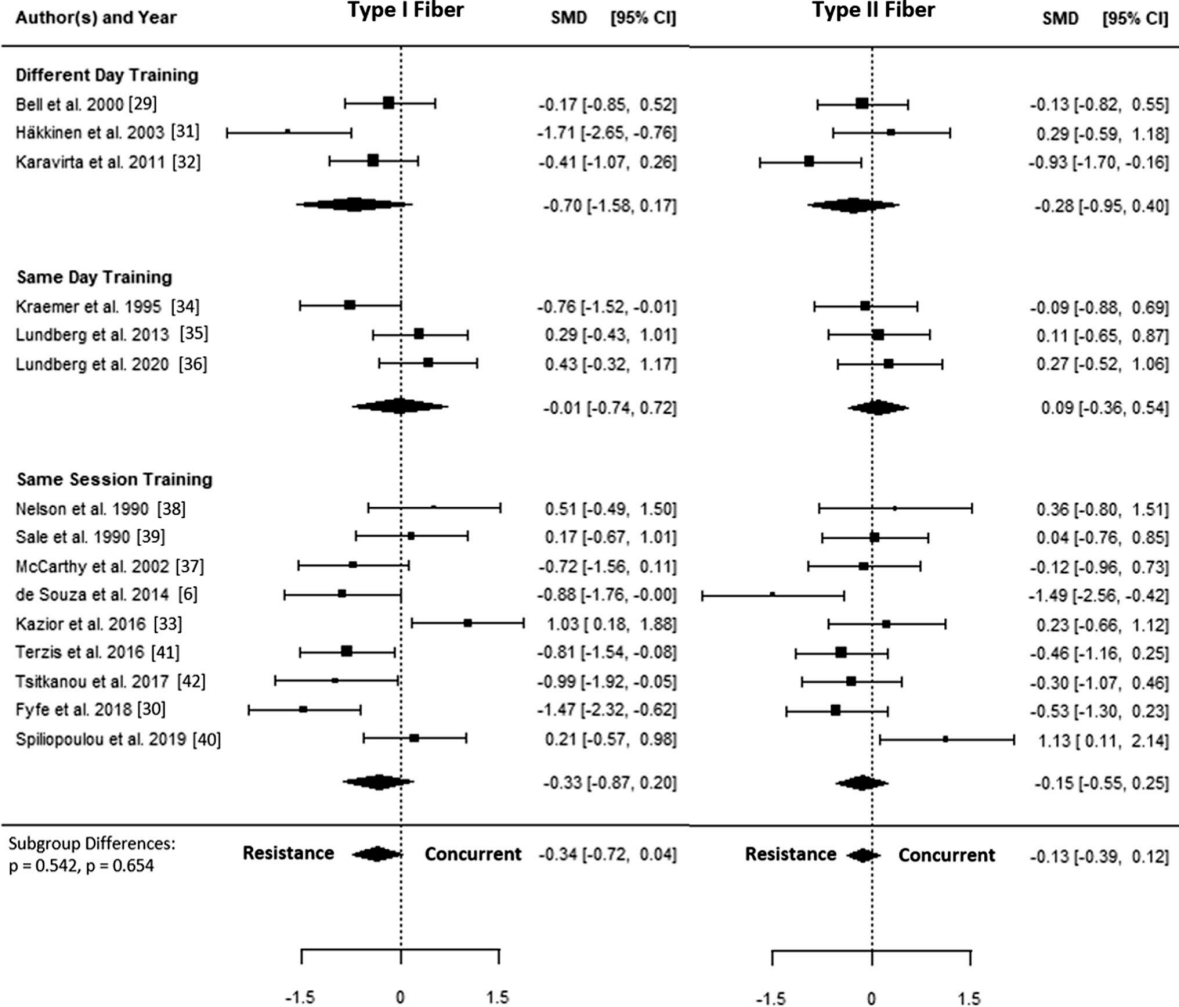
Forest plot comparing differences in hypertrophy of type I and II fibers between different day training, same day training, and same session training. *CI* confidence interval, *SMD* standardized mean difference

**Fig. 7:**
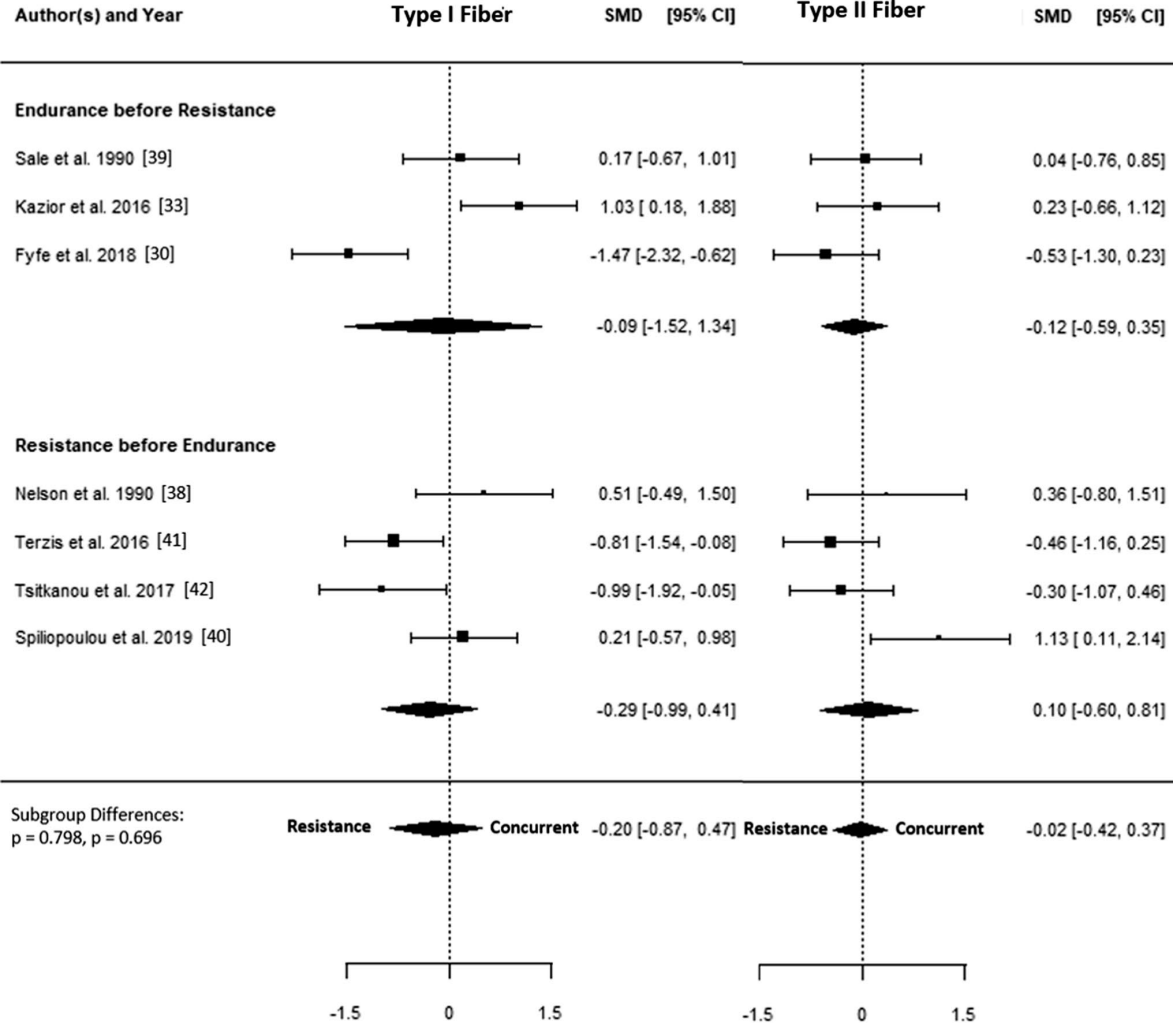
Forest plot comparing differences in hypertrophy of type I and II fibers between different exercise orders during same-session training. *CI* confidence interval, *SMD* standardized mean difference

The SMD for type I fiber hypertrophy ranged from − 1.71 to 1.03. The estimated SMD for the random-effects model was − 0.34 (95% CI − 0.72 to 0.04, *p* = 0.078). The *Q* test revealed that the true outcomes appear to be heterogeneous (*Q*(14) = 44.96, *p* < 0.001, *τ*^2^ = 0.40, *I*^2^ = 70.4%). Of all selected predictors, the number of concurrent training sessions accounted for 34.6% of the observed heterogeneity, while the type of aerobic training (i.e., cycling, running) accounted for 7.3% of the heterogeneity. Subgroup analyses revealed no statistically significant differences between groups (Figs. 3, 4, 5, 6, 7; Figs. S2–S6 of the ESM).

### Type II Muscle Fiber Hypertrophy

The SMD for type II fiber hypertrophy ranged from − 1.49 to 1.13. The estimated SMD based on the random-effects model was − 0.13 (95% CI − 0.39 to 0.12, *p* = 0.315). The *Q* test for heterogeneity was not significant (*Q*(14) = 22.04, *p* = 0.078, *τ*^2^ = 0.08, *I*^2^ = 29.7%). Subgroup analyses revealed no statistically significant differences between groups (Figs. 3, 4, 5, 6, 7; Figs. S7–S11 of the ESM). Furthermore, between-group analyses comparing type I and II fiber hypertrophy within each subgroup revealed no statistically significant differences (*p* ≥ 0.05).

## Discussion

The aim of this study was to evaluate the compatibility of concurrent aerobic and strength training in relation to muscle fiber hypertrophy adaptations. Concurrent training resulted in a small (SMD − 0.2, *p* = 0.05) attenuation of muscle fiber hypertrophy when both type I and type II fibers were combined. The observed heterogeneity was significant only for type I fibers and this was partially explained by the overall training frequency and the type of aerobic training. Indeed, a significant interference effect was observed for type I fibers when aerobic training was performed by running but not cycling. None of the other subgroup analyses (i.e., based on concurrent training frequency, training status, training modality, and training order of same-session training) revealed a statistically significant interference effect for fiber hypertrophy.

Considering that our previous analysis showed no interference effect on whole muscle hypertrophy [4], our finding of interference in muscle fiber size adaptation is intriguing. Because the gold standard measures of muscle size, i.e., MRI and CT, measure the anatomical cross-sectional area of the whole muscle, it is possible that early changes in fiber size are associated with changes in muscle architecture, such as increased pennation angle, mask early hypertrophic effects that are not detected on MRI or CT. Although both aerobic and strength training affect pennation angle [44], few studies have examined the effects of concurrent training on muscle architecture. While Shamim et al. found no differences in the change in pennation angle with concurrent training compared to strength training alone [44], others found a significant increase after concurrent, but not sole, strength training [43]. Thus, while it is still unclear whether architectural changes actually contribute to explaining the discrepancy between fiber and whole muscle hypertrophy, a preferential increase in pennation angle with concurrent training could result in an increased physiological cross-sectional area that is not detected when an anatomical cross-sectional area is assessed on MRI or CT.

Another possibility could relate to the recruitment pattern of each fiber type. This is partly supported by the results of the current study, as the interference effect seems to be mainly owing to the less significant adaptations in type I fibers compared with type II fibers (SMD − 0.3 vs − 0.1), as was also shown by a larger heterogeneity in type I fibers. On the basis of the size principle of motor unit recruitment, endurance training at low-to-moderate intensity recruits mainly type I fibers, whereas at greater intensity (or higher effort level) the recruitment of type II fibers may successively increase [45]. Thus, it is possible that interference induced by aerobic training mainly affects type I fibers, at least during short-term training periods. This could also explain why the observed heterogeneity was lower in type II fibers compared with type I fibers. Considering that type I fibers typically account for a relatively smaller total area of muscle compared with type II fibers in the thigh [46], this supports the idea that it may take longer to detect interference effects at the whole muscle level compared with the fiber level. In this regard, it should be noted that in the study by de Souza et al. that examined both fiber size and whole muscle adaptations, interference in muscle fiber hypertrophy was not confirmed at the whole muscle level [6].

Another interesting finding was that running seemed to exacerbate the interference effect in type I fibers compared with cycling. This could be attributed to the different nature of running compared to cycling, as running is associated with repetitive eccentric loading and stretch–shortening activities, whereas cycling provokes a higher emphasis on concentric work and a longer time under tension. This may, in turn, be associated with greater inflammatory stress induced by running as compared with cycling [47], possibly increasing redox and metabolic stress that may blunt the responsiveness to strength exercise. A direct comparison of muscle hypertrophy induced by aerobic training confirms that cycling can indeed elicit increased muscle size, whereas the effect of running is negligible [48, 49]. However, in the present meta-analysis, the effects of running were based on only three studies. Although caution should be exercised when interpreting these findings, it can be recommended that athletes and fitness enthusiasts seeking to increase muscle mass should consider cycling rather than running as an aerobic training modality.

We also note that many of the studies included in this analysis were small and that the reliability of muscle fiber size measurements is rather low. A recent study examined the variability of histochemical measurements of muscle fiber within subjects and found a coefficient of variation of 13% for fiber area measurements [46]. The authors concluded that if fiber hypertrophy is of particular interest, it is preferable to analyze and average two or more biopsies from different sites to obtain more reliable results. Overall, this demonstrates that caution should be exercised when interpreting muscle fiber size data from single biopsies. It should also be noted that the majority of included studies were of only moderate quality, while two studies were of poor quality and only one was of good quality. Thus, future studies with adequate power, including both whole muscle and fiber measurements from two biopsy sites, may shed further light on the potential difference in the time course of hypertrophic effects of concurrent training and sole strength training.

## Conclusions

Our results suggest that concurrent aerobic and strength training may induce attenuated muscle fiber hypertrophy compared with strength training alone, but this does not necessarily translate into differences in whole muscle hypertrophy. Furthermore, we provide preliminary evidence that this interference effect may be more pronounced when aerobic training is performed by running compared with cycling, at least in type I fibers. Future studies are needed to elucidate possible differences in the time course of the hypertrophic effect at the whole muscle and fiber levels, and to clarify whether there are fiber type-specific effects of concurrent training.

## Supplementary Information

Below is the link to the electronic supplementary material.

Supplementary file1 (PDF 2770 kb)

## Declarations

### Code availability

The code will be available upon reasonable request.

## References

[1] Bull FC, Al-Ansari SS, Biddle S (2020). World Health Organization 2020 guidelines on physical activity and sedentary behaviour. Br J Sports Med.

[2] Hickson RC (1980). Interference of strength development by simultaneously training for strength and endurance. Eur J Appl Physiol Occup Physiol.

[3] Hawley JA (2009). Molecular responses to strength and endurance training: are they incompatible?. Appl Physiol Nutr Metab.

[4] Schumann M, Feuerbacher JF, Sünkeler M (2022). Compatibility of concurrent aerobic and strength training for skeletal muscle size and function: an updated systematic review and meta-analysis. Sports Med.

[5] Craig BW, Lucas J, Pohlman R (1991). The effects of running, weightlifting and a combination of both on growth hormone release. J Strength Cond Res.

[6] de Souza EO, Tricoli V, Aoki MS (2014). Effects of concurrent strength and endurance training on genes related to myostatin signaling pathway and muscle fiber responses. J Strength Cond Res.

[7] Tesch PA (1988). Skeletal muscle adaptations consequent to long-term heavy resistance exercise. Med Sci Sports Exerc.

[8] Hoppeler H, Baum O, Lurman G (2011). Molecular mechanisms of muscle plasticity with exercise. Compr Physiol.

[9] Coffey VG, Hawley JA (2017). Concurrent exercise training: do opposites distract?. J Physiol.

[10] Baar K (2006). Training for endurance and strength: lessons from cell signaling. Med Sci Sports Exerc.

[11] Fyfe JJ, Bishop DJ, Stepto NK (2014). Interference between concurrent resistance and endurance exercise: molecular bases and the role of individual training variables. Sports Med.

[12] Babcock L, Escano M, D'Lugos A (2012). Concurrent aerobic exercise interferes with the satellite cell response to acute resistance exercise. Am J Physiol Regul Integr Comp Physiol.

[13] Petré H, Hemmingsson E, Rosdahl H (2021). Development of maximal dynamic strength during concurrent resistance and endurance training in untrained, moderately trained, and trained individuals: a systematic review and meta-analysis. Sports Med.

[14] Gergley JC (2009). Comparison of two lower-body modes of endurance training on lower-body strength development while concurrently training. J Strength Cond Res.

[15] Jones TW, Howatson G, Russell M (2016). Performance and endocrine responses to differing ratios of concurrent strength and endurance training. J Strength Cond Res.

[16] Liberati A, Altman DG, Tetzlaff J (2009). The PRISMA statement for reporting systematic reviews and meta-analyses of studies that evaluate health care interventions: explanation and elaboration. PLoS Med.

[17] Drevon D, Fursa SR, Malcolm AL (2017). Intercoder reliability and validity of WebPlotDigitizer in extracting graphed data. Behav Modif.

[18] Viechtbauer W (2010). Conducting meta-analyses in R with the metafor package. J Stat Softw.

[19] Morris SB (2008). Estimating effect sizes from pretest-posttest-control group designs. Org Res Methods.

[20] Becker BJ (1988). Synthesizing standardized mean-change measures. Br J Math Stat Psychol.

[21] Viechtbauer W (2005). Bias and efficiency of meta-analytic variance estimators in the random-effects model. J Educ Behav Stat.

[22] Cochran WG (1954). The combination of estimates from different experiments. Biometrics.

[23] Higgins JPT, Thompson SG (2002). Quantifying heterogeneity in a meta-analysis. Stat Med.

[24] Higgins JPT, Green S (2011). Cochrane handbook for systematic reviews of interventions, version 5.1.0.

[25] Viechtbauer W, Cheung MW-L (2010). Outlier and influence diagnostics for meta-analysis. Res Synth Methods..

[26] Begg CB, Mazumdar M (1994). Operating characteristics of a rank correlation test for publication bias. Biometrics.

[27] Rothstein HR, Sutton AJ, Borenstein M (2005). Publication bias in meta-analysis: prevention, assessment and adjustments.

[28] Higgins JP, Green S. Cochrane handbook for systematic reviews of interventions, version 5.1.0. Cochrane Collaboration; 2011.

[29] Elkins MR, Herbert RD, Moseley AM (2010). Rating the quality of trials in systematic reviews of physical therapy interventions. Cardiopulm Phys Ther J.

[30] Bell GJ, Syrotuik D, Martin TP (2000). Effect of concurrent strength and endurance training on skeletal muscle properties and hormone concentrations in humans. Eur J Appl Physiol.

[31] Fyfe JJ, Bishop DJ, Bartlett JD (2018). Enhanced skeletal muscle ribosome biogenesis, yet attenuated mTORC1 and ribosome biogenesis-related signalling, following short-term concurrent versus single-mode resistance training. Sci Rep.

[32] Häkkinen K, Alen M, Kraemer WJ (2003). Neuromuscular adaptations during concurrent strength and endurance training versus strength training. Eur J Appl Physiol.

[33] Karavirta L, Häkkinen A, Sillanpää E (2011). Effects of combined endurance and strength training on muscle strength, power and hypertrophy in 40–67-year-old men. Scand J Med Sci Sports.

[34] Kazior Z, Willis SJ, Moberg M (2016). Endurance exercise enhances the effect of strength training on muscle fiber size and protein expression of Akt and mTOR. PLoS ONE.

[35] Kraemer WJ, Patton JF, Gordon SE, et al. Compatibility of high-intensity strength and endurance training on hormonal and skeletal muscle adaptations. J Appl Physiol (1985). 1995;78(3):976–89. 10.1152/jappl.1995.78.3.976 7775344

[36] Lundberg TR, Fernandez-Gonzalo R, Gustafsson T (2013). Aerobic exercise does not compromise muscle hypertrophy response to short-term resistance training. J Appl Physiol (1985).

[37] Lundberg TR, Martínez-Aranda LM, Sanz G (2020). Early accentuated muscle hypertrophy is strongly associated with myonuclear accretion. Am J Physiol Regul Integr Comp Physiol.

[38] McCarthy JP, Pozniak MA, Agre JC (2002). Neuromuscular adaptations to concurrent strength and endurance training. Med Sci Sports Exerc.

[39] Nelson AG, Arnall DA, Loy SF (1990). Consequences of combining strength and endurance training regimens. Phys Ther.

[40] Sale DG, MacDougall JD, Jacobs I (1990). Interaction between concurrent strength and endurance training. J Appl Physiol (1985).

[41] Spiliopoulou P, Zaras N, Methenitis S (2021). Effect of concurrent power training and high-intensity interval cycling on muscle morphology and performance. J Strength Cond Res.

[42] Terzis G, Spengos K, Methenitis S (2016). Early phase interference between low-intensity running and power training in moderately trained females. Eur J Appl Physiol.

[43] Tsitkanou S, Spengos K, Stasinaki A-N (2017). Effects of high-intensity interval cycling performed after resistance training on muscle strength and hypertrophy. Scand J Med Sci Sports.

[44] Shamim B, Devlin BL, Timmins RG (2018). Adaptations to concurrent training in combination with high protein availability: a comparative trial in healthy, recreationally active men. Sports Med.

[45] Henneman E (1985). The size-principle: a deterministic output emerges from a set of probabilistic connections. J Exp Biol.

[46] Horwath O, Envall H, Röja J (2021). Variablity in vastus lateralis fiber type distribution, fiber size and myonuclear content along and the between legs. J Appl Physiol (1985).

[47] Nieman DC, Luo B, Dréau D (2014). Immune and inflammation responses to a 3-day period of intensified running versus cycling. Brain Behav Immun.

[48] Grgic J, Mcllvenna LC, Fyfe JJ (2019). Does aerobic training promote the same skeletal muscle hypertrophy as resistance training? A systematic review and meta-analysis. Sports Med.

[49] Konopka AR, Harber MP (2014). Skeletal muscle hypertrophy after aerobic exercise training. Exerc Sport Sci Rev.

