# Supplementary material for: The Effects of Concurrent Aerobic and Strength Training on Muscle Fiber Hypertrophy: A Systematic Review and Meta-Analysis
Source: Sports Med. 2022 Apr 27;52(10):2391–403. doi: 10.1007/s40279-022-01688-x (PMC9474354; doi:10.1007/s40279-022-01688-x)
Supplement: Supplementary file 1 — Supplementary file1 (PDF 2770 kb) [file 40279_2022_1688_MOESM1_ESM.pdf]

Electronic Supplementary Material

Table S1: Databases and the corresponding search string.

Database	Search string
PubMed/MEDLINE	(("Humans"[MeSH Terms] AND (("concurrent"[All Fields] OR "combin*"[All Fields]) OR "simultaneous"[All Fields])) AND ("strength"[All Fields] OR "resistance"[All Fields])) AND ("endurance"[All Fields] OR "aerobic"[All Fields])
ISI Web of Science	TS=Humans OR people AND concurrent training OR combined training AND strength exercise OR resistance exercise OR resistance training OR strength training AND aerobic training OR aerobic exercise OR endurance training OR endurance exercise
Embase	TS=Humans OR people AND concurrent training OR combined training AND strength exercise OR resistance exercise OR resistance training OR strength training AND aerobic training OR aerobic exercise OR endurance training OR endurance exercise
CINAHL	TS=Humans OR people AND concurrent training OR combined training AND strength exercise OR resistance exercise OR resistance training OR strength training AND aerobic training OR aerobic exercise OR endurance training OR endurance exercise
SPORTDiscus	TS=Humans OR people AND concurrent training OR combined training AND strength exercise OR resistance exercise OR resistance training OR strength training AND aerobic training OR aerobic exercise OR endurance training OR endurance exercise
Scopus	TS=Humans OR people AND concurrent training OR combined training AND strength exercise OR resistance exercise OR resistance training OR strength training AND aerobic training OR aerobic exercise OR endurance training OR endurance exercise

Table S2: PEDro Scores for the included studies.

Authors (year)	PEDro Scale criterion number											Total
	1	2	3	4	5	6	7	8	9	10	11	
Bell et al. (2000) [1]	No	1	0	1	0	0	0	0	0	1	1	**4**
de Souza et al. (2014) [2]	Yes	1	0	1	0	0	0	1	0	1	1	**5**
Fyfe et al. (2018) [3]	No	1	0	1	0	0	0	0	0	1	1	**4**
Häkkinen et al. (2003) [4]	No	0	0	1	0	0	0	0	0	1	1	**3**
Karavirta et al. (2011) [5]	Yes	1	0	1	0	0	0	1	0	1	1	**5**
Kazior et al. (2016) [6]	No	0	0	1	0	0	0	0	0	1	1	**3**
Kraemer et al. (1995) [7]	No	1	1	1	0	0	0	1	0	1	1	**6**
Lundberg et al. (2013) [8]	No	1	0	1	0	0	0	1	0	1	1	**5**
Lundberg et al. (2020) [8][8]	No	0	0	1	0	0	0	1	0	1	1	**4**
McCarthy et al. (2002) [9]	No	1	0	1	0	0	0	1	0	1	1	**5**
Nelson et al. (1990)	No	1	0	1	0	0	0	0	0	1	1	**4**
Sale et al. (1990) [10]	No	1	0	1	0	0	0	0	0	1	1	**4**
Spiliopoulou et al. (2019) [11]	No	0	0	1	0	0	0	1	0	1	1	**4**
Terzis et al. (2016) [12]	No	1	0	1	0	0	0	1	0	1	1	**5**
Tsitkanou et al. (2017) [13]	No	0	0	1	0	0	0	1	0	1	1	**4**

1 = eligibility criteria were specified, 2 = subjects were randomly allocated to groups, 3 = allocation was concealed, 4 = groups were similar at baseline, 5 = all subjects were blinded, 6 = therapist who administered therapy/training were blinded, 7 = all assessors who measured key outcomes were blinded, 8 = measurement of key outcomes were obtained from more than 85% of the subjects, 9 = subjects for whom outcome measures were available received the treatment or control condition as allocated or, otherwise for at least one key outcome was analysed by "intention to treat", 10 = results of between-group statistical comparisons were reported for at least on key outcome, 11 = study provides both point measures and measures of variability, score: 0 = no, 1 = yes.

Table S3: Characteristics of included studies.

Author(s)	Participants	Training modality		Strength Training	Aerobic Training	Outcome
Bell et al. (2000) [1]	ST = 11, CT = 13; physically active university students	12 weeks, ST: 3 sessions/week CT: 6 sessions/week (3 × ST + 3 × AT)	Different day CT, ST and AT performed on alternating days	Exercise: single leg - leg press, leg flexion/extension, calf raises, bench press, latissimus-pull, shoulder press, biceps curls<br>Set configuration: 2-6 × 4-12 repetitions at 72-84% 1RM intensity increased by 4% every 3 weeks,	Cycling: continuous cycling at 30 min per session and progressed to 42 min per session (4 min increase every 4 weeks), interval sessions were performed once a week, 4-6 × 3 min at 90% $\dot{V}O_2$max, and 3 min of active recovery between each bout (increased by 1 set every 4 weeks)	Type I Fiber ↑[a]<br>Type II Fiber ↑[a b]
de Souza et al. (2014) [2]	ST = 11, CT = 11; active physical education students	8 weeks, ST: 2 sessions/week CT: 4 sessions/week (2 × ST + 2 × AT)	Same session CT, exercise order was altered during training	Exercise: knee extension, knee flexion, leg-press 45°, knee extension and knee flexion<br>Set configuration: 3-5 × 6-12 repetitions	Running: intensity was 80-100% of the maximal velocity at $\dot{V}$O2max; 15-20 × of 60 sec b1outs with 45-90 sec rest	Type I Fiber ↑ [a]<br>Type II Fiber ↑ [a]<br>(no significant increase in Type IIx)
Fyfe et al. (2018) [3]	ST = 8, HIT+ST = 8, MICT+ST = 7; recreationally active	8 weeks, ST: 2 sessions/week CT: 6 sessions/week (3 × ST + 3 × AT)	Same session CT, AT performed 10 min before ST	Exercise: one session: leg press, bench press, seated row, leg extension and leg curl exercises were included; other session: leg press, flat dumbbell press, latissimus pulldown, dumbbell lunges and leg curl exercises<br>Set configuration: 3-5 × 4-14 repetitions at 65-90% 1RM	Cycling: HIT: 2 min intervals at intensities between 120 and 150% of the lactate threshold (LT) and 1 min recovery; MICT: 15 - 33 min at a relative intensity between 80 and 100% of the LT	Type I Fiber ↑ [a c] (only for ST vs. HIT + ST)<br>Type II Fiber →
Häkkinen et al. (2003) [4]	ST = 16, CT = 11; healthy	21 weeks, ST: 2 sessions/week CT: 4 sessions/week (2 × ST + 2 × AT)	Different day CT, |ET and ST performed on separate days	Exercise: leg press, bilateral/unilateral knee extension, bench press, triceps push, latissimus pull, sit-up, trunk extensor, elbow	Cycling or walking; weeks 1-7, 30 min of continuous cycling or walking below aerobic threshold level; weeks 8-14, 45 min, including 15 min below the aerobic threshold, 10 min between aerobic-anaerobic thresholds, 5 min above the	Type I Fiber ↑[a b]<br>Type II Fiber ↑[a b]

				flexion, leg abduction/adduction Set configuration: week 1-7, 3-4 × 10-15 repetitions at 50-70% 1RM, weeks 8-14, 3-5 × 5-6 repetitions at 60-80% 1RM and 3-5 × 8-12 repetitions at 50-60% 1RM for leg extensor exercises and 3-5 × 10-12 repetitions for all other exercises, weeks 15-21 subjects performed either higher loads with 4-6 × 3-6 repetitions at 70-80% 1RM and loads with 4-6 × 8-12 repetitions at 50-60% 1RM for leg extensor exercises and 3-5 × 8-12 repetitions for all other exercises	anaerobic threshold and 15 min again under the aerobic threshold or 60 min continuous work below aerobic threshold; weeks 15 - 21, 60 min of exercise including 15 min under the aerobic threshold, 2 × 10 min between aerobic-anaerobic thresholds, 2 × 5 min above anaerobic threshold and the final 15 min under aerobic threshold or 60-90 min continuous work below aerobic threshold	
Karavirta et al. (2011) [5]	ST = 25, CT = 25; untrained	8 weeks, ST: 2 sessions/week CT: 4 sessions/week (2 × ST + 2 × AT)	Different day CT, ST and AT performed on separate days	Exercise: leg press, bilateral/unilateral knee extension, bench press, triceps push, latissimus pull, sit-up, trunk extensor, elbow flexion, leg abduction/adduction Set configuration: week 1-7, 3-4 × 10-15 repetitions at 50-70% 1RM, weeks 8-14, 3-5 × 5-6 repetitions at 60-80% 1RM and 3-5 × 8-12 repetitions at 50-60% 1RM for leg extensor exercises and 3-5 ×	Cycling: weeks 1-7, continuous bicycle ergometer for 30 min below aerobic threshold; in addition, during weeks 5-7, subjects did three sessions,10 min interval with intensity above aerobic threshold; weeks 8-14, either 45 min of exercise including 10 min of work between the aerobic-anaerobic thresholds and 5 min above anaerobic threshold, in addition to 15 min warm up and 15 min cool down below aerobic threshold or 60 min continuous work below aerobic threshold; weeks 15-21, 60 min of exercise including 2 × 10 min intervals between the aerobic-anaerobic thresholds, 2 × 5 min of work above anaerobic threshold and 30 min below the aerobic threshold or 90 min	Type I Fiber ↑ [a] Type II Fiber ↑

				10-12 repetitions for all other exercises, weeks 15-21 subjects performed either higher loads with 4-6 × 3-6 repetitions at 70-80% 1RM and loads with 4-6 × 8-12 repetitions at 50-60% 1RM for leg extensor exercises and 3-5 × 8-12 repetitions for all other exercises	continuous work below aerobic threshold	
Kazior et al. (2016) [6]	ST = 7, CT = 9; healthy	7 weeks<br>ST: 2-4 sessions/week<br>CT: 4-8 sessions/week<br>(2-4 × ST + 2-4 × AT)	Same session CT, AT was performed before ST training	Exercise: leg press, Set configuration: 70% of 1RM and this load was raised 5–7% every third or fourth training session, The number of × was increased from four at week one to six at week five and number of repetitions in each set decreased from twelve to eight with a 3-min rest between sets. The subjects were guided to perform each repetition at a set pace, i.e., with concentric and eccentric phases of 2 seconds each	Cycling: ergometer cycling at 63 ± 1.2% of $\dot{V}O_2max$ with training intensity being increased progressively every two weeks; interval cycling at 95 ± 1.8% of $\dot{V}O_2max$ in the final three weeks	Type I Fiber (↓ for ST; ↑ for CT) [b]<br><br>Type II Fiber ↑ [a b]
Kraemer et al. (1995) [7]	ST = 9, CT1 = 9, CT2 = 9; healthy	12 weeks,<br>ST: 4 sessions/week<br>CT: 8 sessions/week<br>(4 × ST + 4 × AT)	Same day CT, AT was performed before ST after 5 to 6 hours rest	Exercise: various free weight/machine exercises, targeting the major upper- and lower body muscles<br>Set configuration:	Running: long-distance or sprint-interval workouts; long distance training, running as far as possible in 40 min; sprint-interval training, interval distances ranging from 200-800 m and intensities between 95-100% of $\dot{V}O_2max$; exercise-to-rest ratio progressed from 1:4 to 1:0.5	Type I Fiber ↑[a b]<br>Type II Fiber ↑[a b] (only Type IIa Fiber increase in CT Group)

				2-3 × 10-25 repetitions and 3-5 × 5-10 repetitions		
Lundberg et al. (2013) [8]	Same person different limbs<br>n = 10, moderately trained	5 weeks,<br>ST: 2-3 sessions/wk<br>CT: 5-6 sessions/wk (2-3 × ST + 3 × AT)	Same day CT, | AT was performed 6 hours before ST	Exercise: both limbs; Set configuration:<br>4 × 7 maximal knee extensions	Cycling: 40 min continuous one-legged cycle ergometer exercise at 70% of peak power; after 40 min workload increased by ~20 W until failure	Type I Fiber ↑<br>Type II Fiber ↑ [b]
Lundberg et al. (2020) [14]	Same person different limbs<br>n = 10 | recreationally active	5 weeks,<br>ST: 2-3 sessions/wk<br>CT: 5-6 sessions/wk (2-3 × ST + 3 × AT)	Same day CT, AT was performed 6 hours before ST	Exercise: both limbs; Set configuration:<br>4 × 7 maximal knee extensions	Cycling: 40 min continuous one-legged cycle ergometer exercise at 70% of peak power; after 40 min workload increased by ~20 W until failure	Type I Fiber ↑[b]<br>Type II Fiber ↑[a b]
McCarthy et al. (2002) [9]	ST = 10, CT = 10; sedentary healthy	10 weeks,<br>ST: 3 sessions/wk<br>CT: 6 sessions/wk (3 × ST + 3 × AT)	Same session CT, AT and ST performed in alternating order	Exercise:<br>squats, bench press, standing curls, knee extension, leg curl, lat. Pull-down, overhead press, heel raise<br>Set configuration: 3 x 6 repetitions with maximal efforts	Cycling: 50 min continuous cycling ergometer exercise at 70% HRR	Type I Fiber ↑[a]<br>Type II Fiber ↑[a b]

Nelson et al. (1990)[15]	same person different limbs<br>n = 10 | untrained healthy	11 weeks,<br>ST: 4 sessions/wk<br>CT: 8 sessions/wk (4 × ST + 4 × AT)	Same session CT, AT was performed 10 minutes after ST	Exercise: both limbs; Set configuration:<br>3 × 6 maximal-effort repetitions of knee extension and flexion	Cycling: 30 - 50 min continuous cycling ergometer exercise at 75 - 85% of the HRmax	Type I Fiber (↓ in ST, ↑ in CT) [b c]<br><br>Type II Fiber ↑ [a b] (in ST significant increase only in Type IIb Fiber)
Sale et al. (1990) [10]	n = 8<br>same person different limb	22 weeks,<br>ST: 3 sessions/wk<br>CT: 6 sessions/wk (3 × ST + 3 × AT)	Same session CT, AT was performed before ST training	Exercise: unilateral leg press;<br>Set configuration 6 × 15-20 repetitions, × were alternated between legs (group A) or performed with the randomly designated leg (group B) with 1-2 min rest in between	Cycling: 5 × of 3 min one-legged cycling on ergometer at 90-100% $\dot{V}O_2max$; training was performed with the assigned leg for endurance (group A) or alternately with both legs (group B) with 1-3 min rest in between	Type I Fiber ↑<br>Type II Fiber ↑
Spiliopoulou et al. (2019) [11]	ST = 10, CT = 10;<br>physical education students	22 weeks,<br>ST: 3 sessions/wk<br>CT: 6 sessions/wk (3 × ST + 3 × AT)	Same session CT, ST was performed before AT	Exercise: 2 of 3 days per week consisted of 6 × 2 fast eccentric-only half squats, with each set followed by 3 CMJs;<br>Set configuration: training load for half-squat exercise was gradually increased from 40% 1RM in weeks 1-2, to 55% 1RM in weeks 3-4, up to 65% 1RM in weeks 5-6; training on the other day included 8 × 3 CMJs and 8 × 3 Drop Jumps (DJ); initial height for DJs was gradually	Cycling: high intensity intervals on stationary bicycle, 10 bouts of 1 min cycling at maximal aerobic power (mean: 141 ± 13 W) with 1 min passive rest; workload increased 5% every week	Type I Fiber ↑[a b]<br><br>Type II Fiber ↑[a b]

				increased from 20 cm in weeks 1-2, to 30 cm in weeks 3-4, to 40 cm in weeks 5-6		
Terzis et al. (2016) [12]	ST = 10, CT = 10; physical education students	6 weeks, ST: 3 sessions/wk CT: 6 sessions/wk (3 × ST + 3 × AT)	Same session CT, ST was performed before AT	Exercise: 2 of 3 days per week consisted of 6 × 2 fast eccentric-only half squats, with each set followed by 3 CMJs; Set configuration: training load for half-squat exercise was gradually increased from 40% 1RM in weeks 1-2, to 55% 1RM in weeks 3-4, up to 65% 1RM in weeks 5-6; training on the other day included 8 × 3 CMJs and 8 × 3 Drop Jumps (DJ); initial height for DJs was gradually increased from 20 cm in weeks 1-2, to 30 cm in weeks 3-4, to 40 cm in weeks 5-6	Running: walking/jogging for 30 min at 60–70% of HRmax; training intensity was gradually increased from initially 1.8 m × s^{-1} to 2.2 m × s^{-1}	Type I Fiber (↑ in ST, ↓ in CT)[a] Type II Fiber (↑ in ST, → in CT)[a]

Tsitkanou et al. (2017) [13]	ST = 11, CT = 10; university students	8 weeks,<br>ST: 2 sessions/wk<br>CT: 4 sessions/wk (2 × ST + 2 × AT)	Same session CT, ST was performed before AT	Exercise: inclined leg press and half squat exercises;<br>Set configuration: 4 × 6 repetitions at 80% of 6 repetitions in the first week, load was increased by 2.0-2.5% in every training; in addition, 2 × 10 repetitions of abdominal crunches, lateral crunches and dorsal raises were performed during warm up prior to training	Cycling: 10 × of 60 seconds at 100% of maximal aerobic power at 55-60 rpm; training load increased by +2%	Type I Fiber ↑[a b]<br><br>Type II Fiber ↑[a b]

ST = strength training, AT = endurance training, CT = concurrent training, 1RM = one repetition maximum, CMJ = countermovement jump, RFD = rate of force-development, HRmax = maximal heart rate, HRR =heart rate reserve, $\dot{V}O_2max$ = maximal oxygen consumption, significant pre vs. post difference in the strength training group, [b] significant pre vs. post difference in the concurrent training group, [c] between-group difference in favour of strength training, statistically significant difference ($p \leq 0.05$).

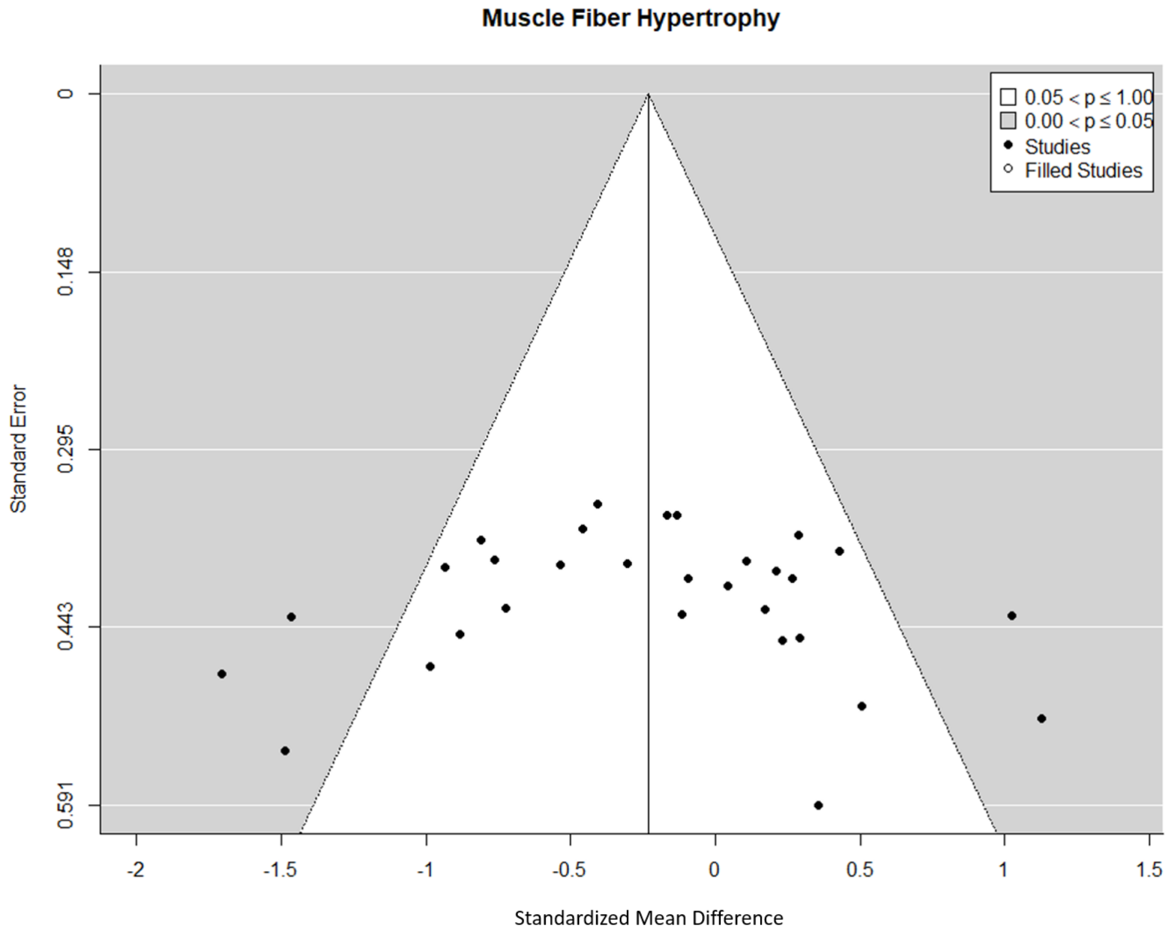

Figure S1. Trim-and-fill funnel plot for muscle fiber hypertrophy. Neither the rank correlation nor the regression test indicated any funnel plot asymmetry (p = 0.860 and p = 0.960, respectively).

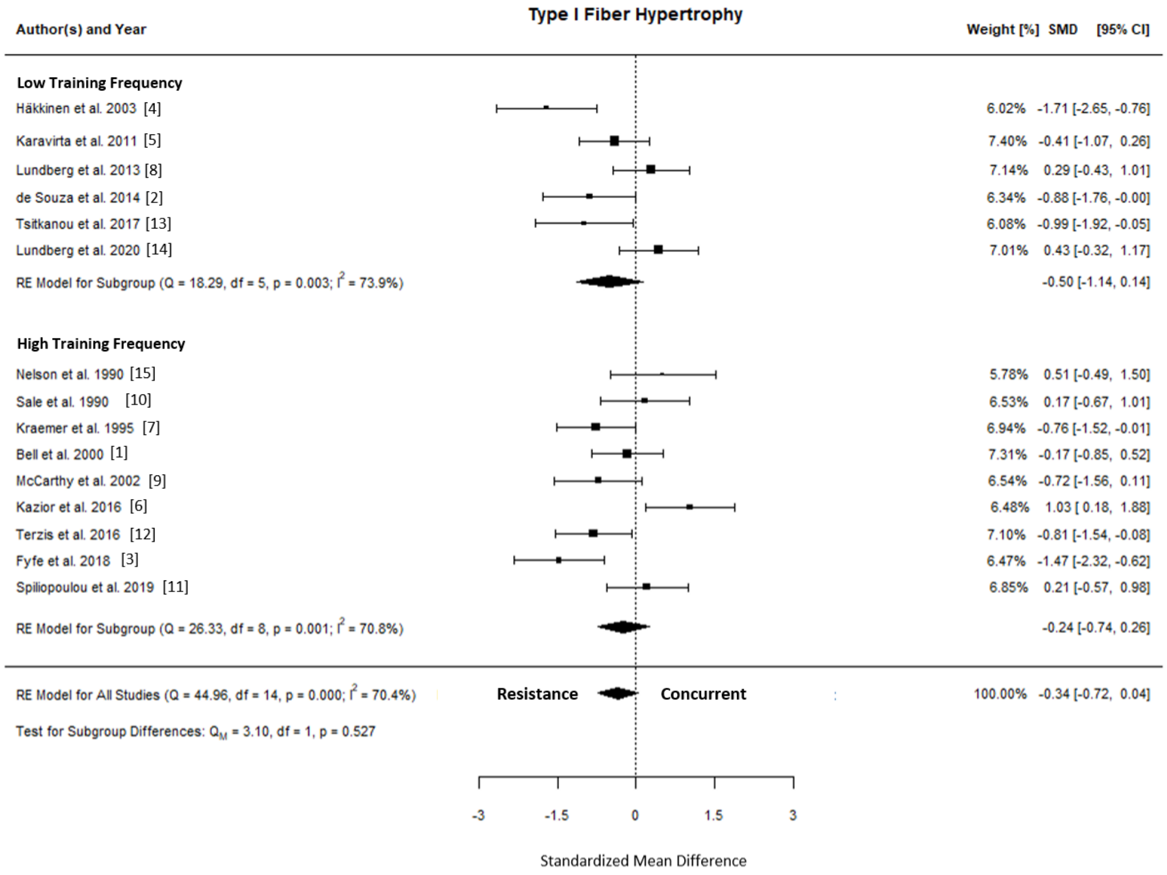

Figure S2. Forest-plot comparing differences in muscle fiber hypertrophy of type I fibers between low and high training frequency. SMD = standardized mean difference, CI = confidence interval, RE = random effects.

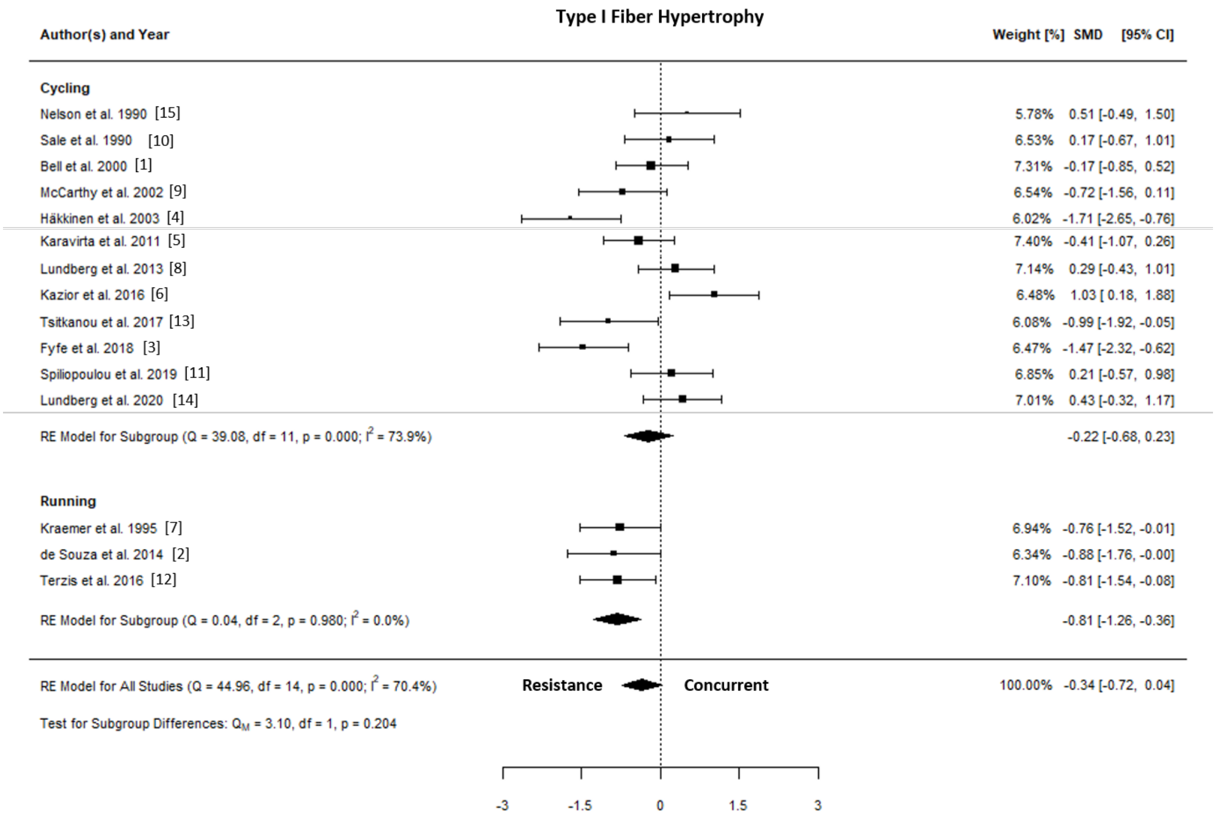

Figure S3. Forest-plot comparing differences in muscle fiber hypertrophy of type I fibers separated by type of aerobic training. SMD = standardized mean difference, CI = confidence interval, RE = random effects.

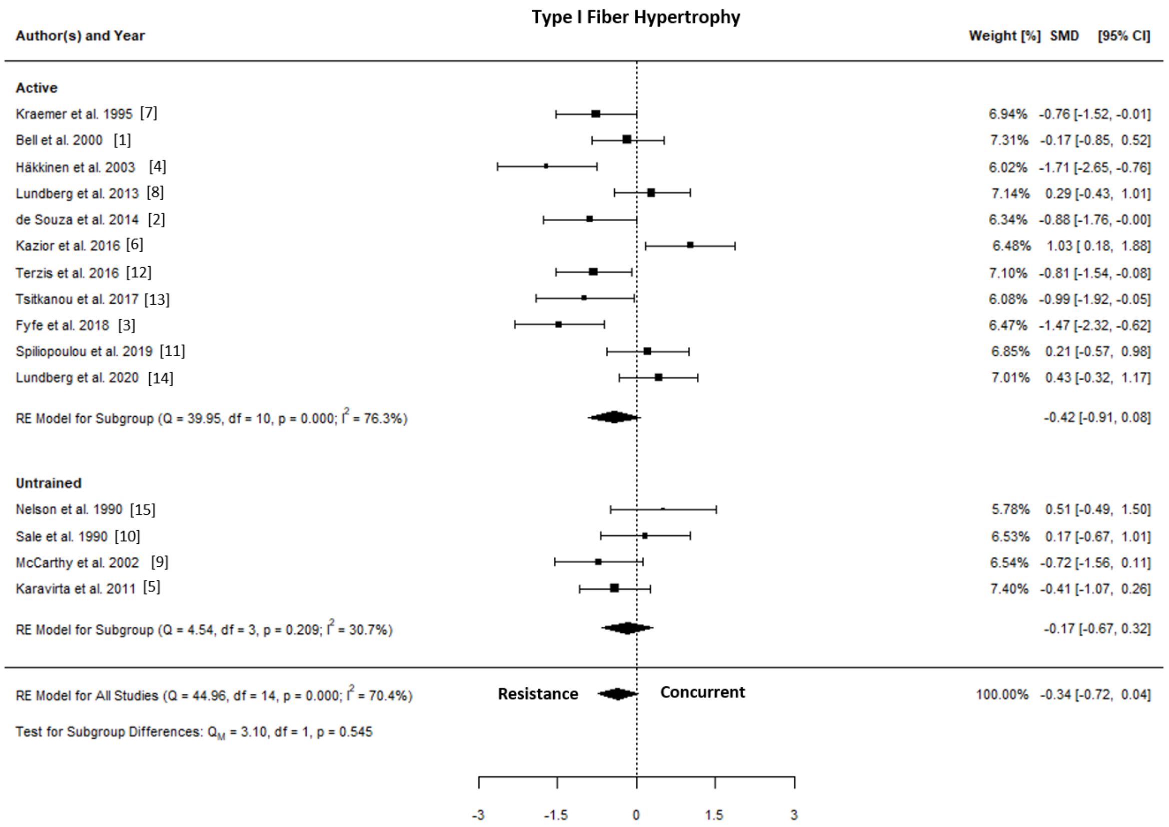

Figure S4. Forest-plot comparing differences in muscle fiber hypertrophy of type I fibers between active and untrained participants. SMD = standardized mean difference, CI = confidence interval, RE = random effects.

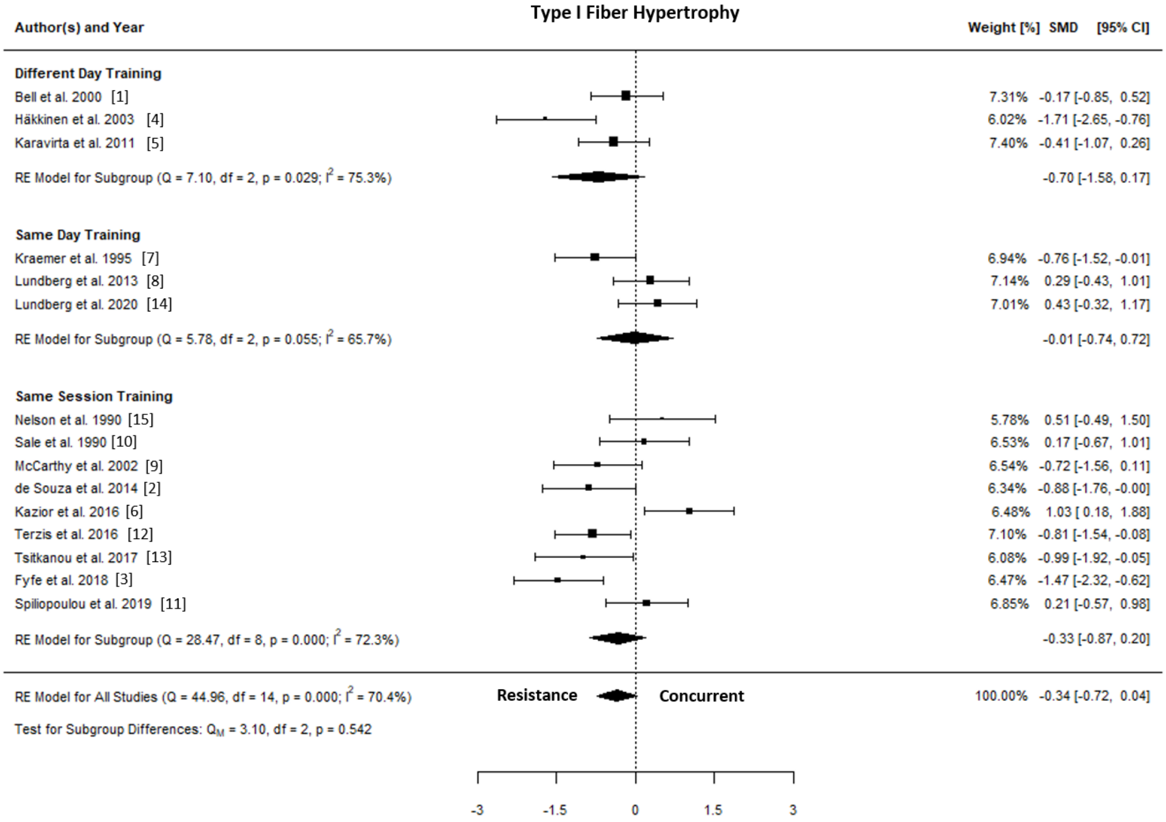

Figure S5. Forest-plot comparing differences in muscle fiber hypertrophy of type I fibers between different day training, same day training and same session training. SMD = standardized mean difference, CI = confidence interval, RE = random effects.

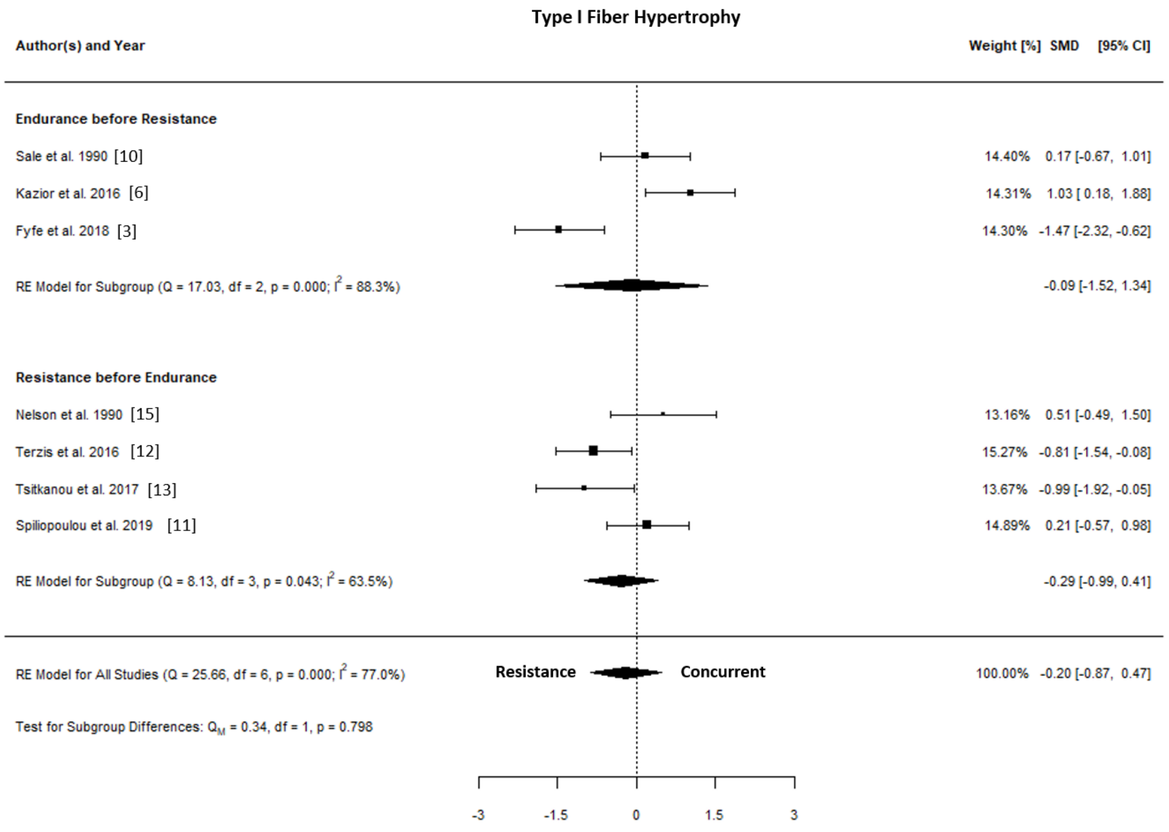

Figure S6. Forest-plot comparing differences in muscle fiber hypertrophy of type I fibers between different exercise order in same session training. SMD = standardized mean difference, CI = confidence interval, RE = random effects.

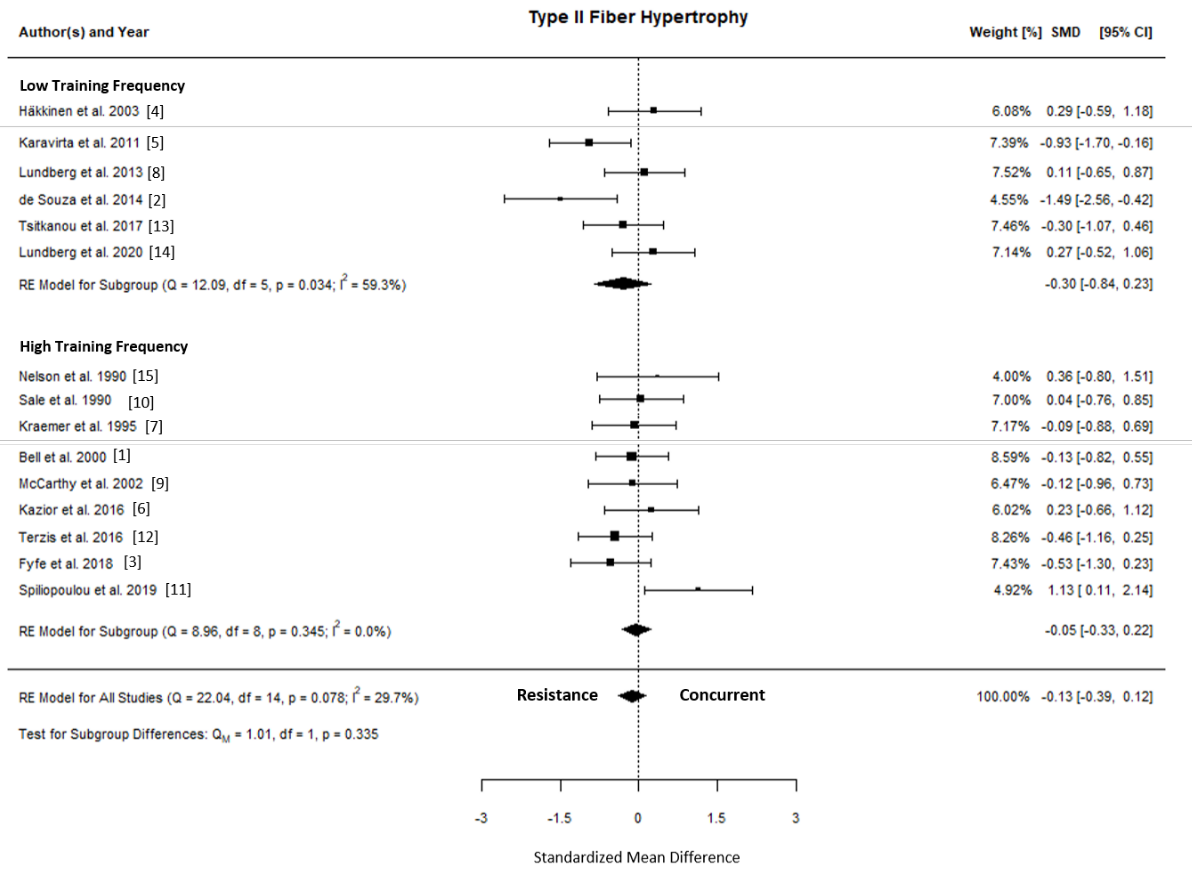

Figure S7. Forest-plot comparing differences in muscle fiber hypertrophy of type II fibers between low and high training frequency. SMD = standardized mean difference, CI = confidence interval, RE = random effects.

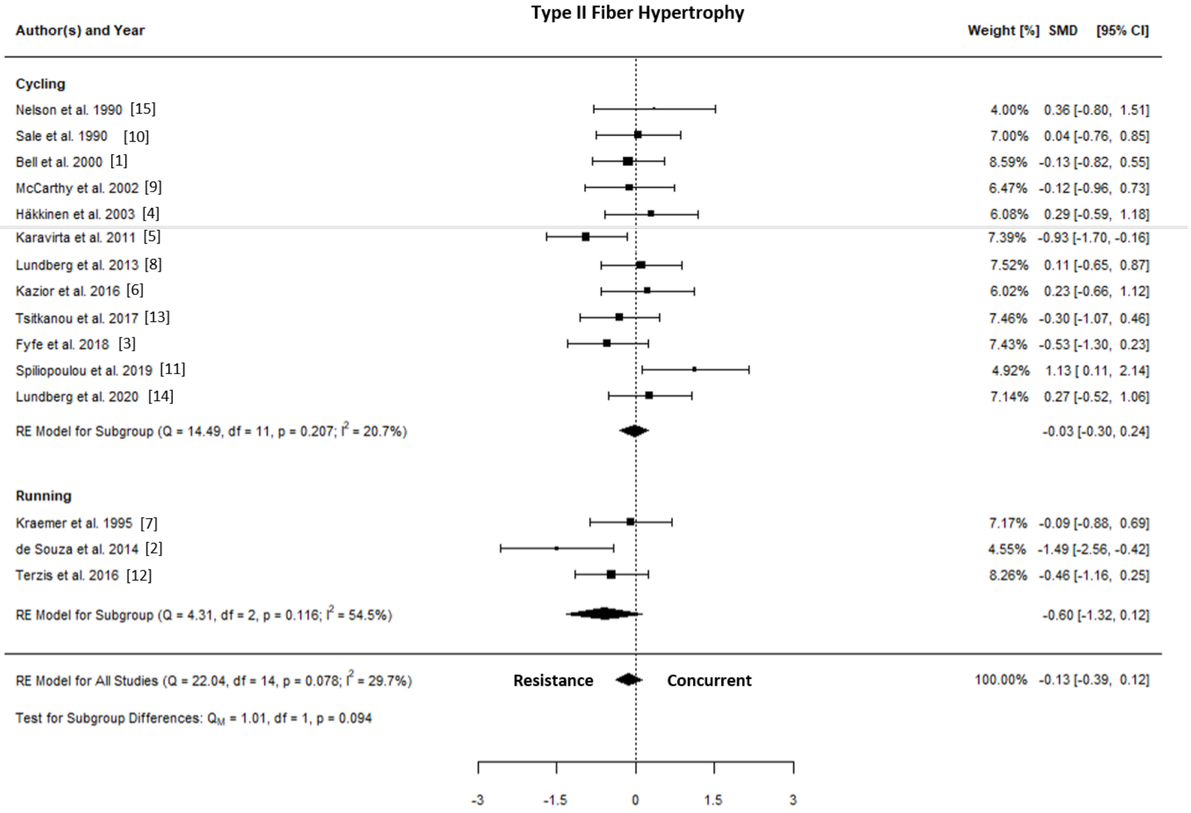

Figure S8. Forest-plot comparing differences in muscle fiber hypertrophy of type II fibers separated by type of aerobic training. SMD = standardized mean difference, CI = confidence interval, RE = random effects.

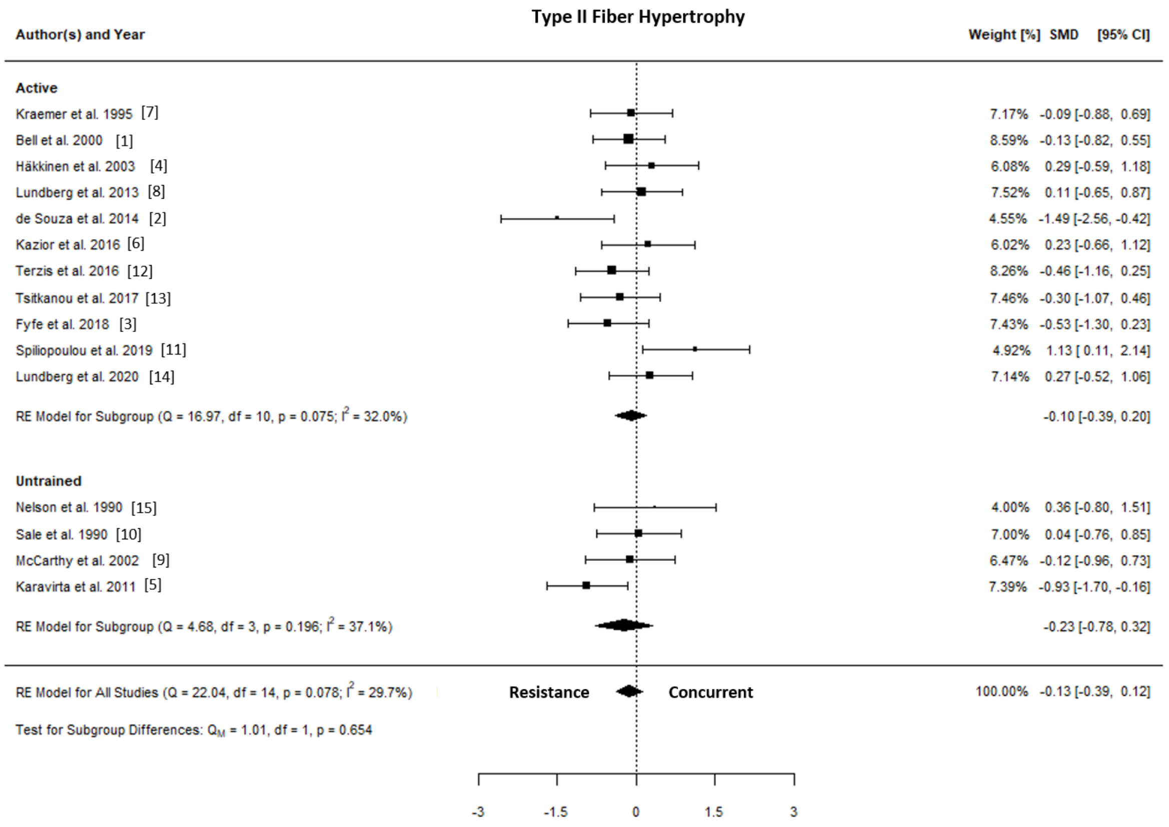

Figure S9. Forest-plot comparing differences in muscle fiber hypertrophy of type II fibers between active and untrained participants. SMD = standardized mean difference, CI = confidence interval, RE = random effects.

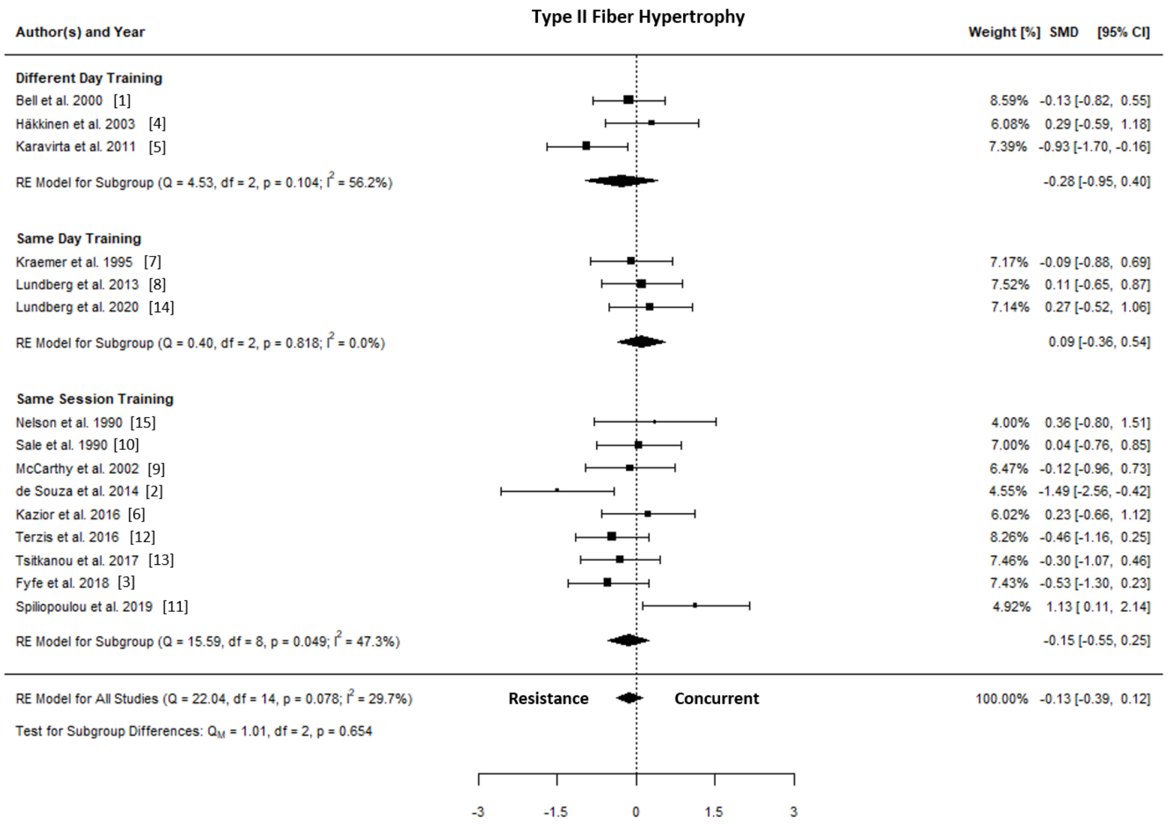

Figure S10. Forest-plot comparing differences in muscle fiber hypertrophy of type II fibers between different day training, same day training and same session training. SMD = standardized mean difference, CI = confidence interval, RE = random effects.

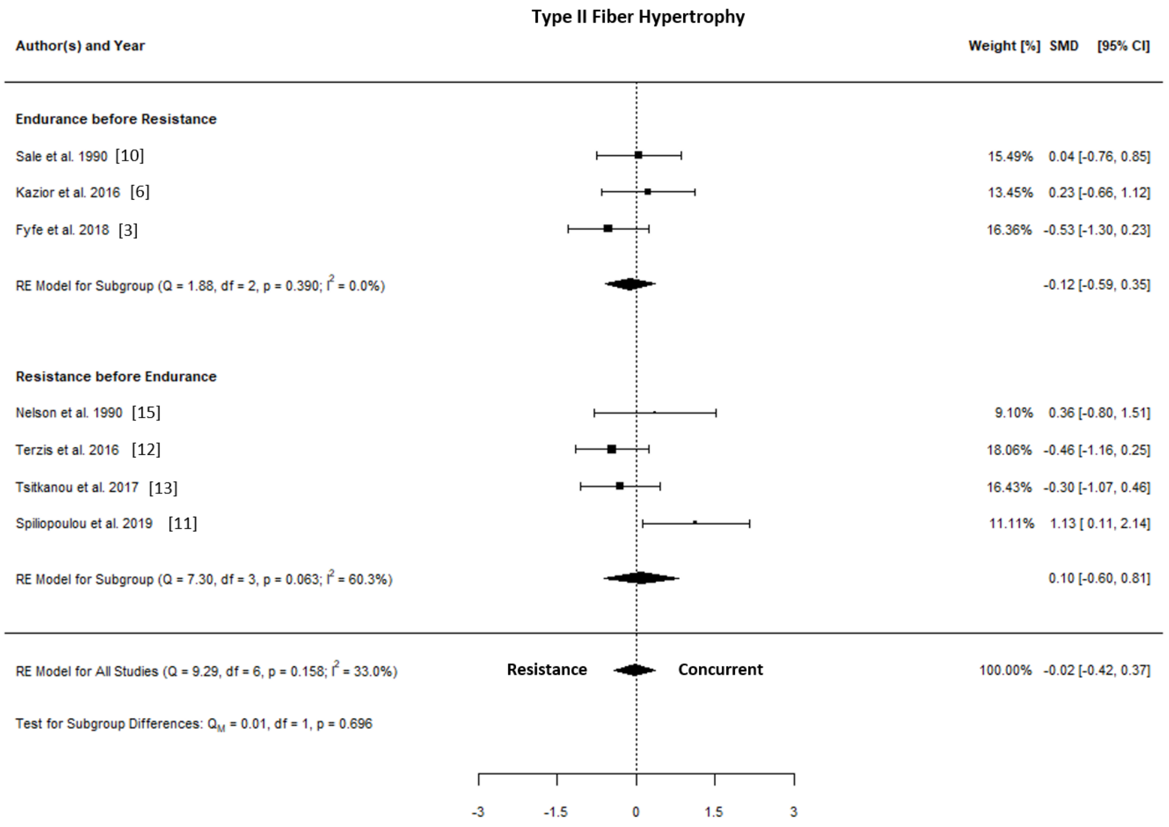

Figure S11. Forest-plot comparing differences in muscle fiber hypertrophy of type II fibers between different exercise order in same session training. SMD = standardized mean difference, CI = confidence interval, RE = random effects.

REFERENCES

1 Bell GJ, Syrotuik D, Martin TP, et al. Effect of concurrent strength and endurance training on skeletal muscle properties and hormone concentrations in humans. *European Journal of Applied Physiology* 2000;81(5):418–27.

2 Souza EO de, Tricoli V, Roschel H, et al. Molecular adaptations to concurrent training. *Int J Sports Med* 2013;34(3):207–13.

3 Fyfe JJ, Bartlett JD, Hanson ED, et al. Endurance training intensity does not mediate interference to maximal lower-body strength gain during short-term concurrent training. *Frontiers in Physiology* 2016;7(NOV). http://www.embase.com/search/results?subaction=viewrecord&from=export&id=L613725387 http://dx.doi.org/10.3389/fphys.2016.00487.

4 Häkkinen K, Alen M, Kraemer WJ, et al. Neuromuscular adaptations during concurrent strength and endurance training versus strength training. *European Journal of Applied Physiology* 2003;89(1):42–52. doi:10.1007/s00421-002-0751-9 [published Online First: 14 December 2002].

5 Karavirta L, Häkkinen A, Sillanpää E, et al. Effects of combined endurance and strength training on muscle strength, power and hypertrophy in 40-67-year-old men. *Scandinavian Journal of Medicine & Science in Sports* 2011;21(3):402–11. doi:10.1111/j.1600-0838.2009.01059.x [published Online First: 18 December 2009].

6 Kazior Z, Willis SJ, Moberg M, et al. Endurance exercise enhances the effect of strength training on muscle fiber size and protein expression of akt and mTOR. *PLoS One* 2016;11(2). http://www.embase.com/search/results?subaction=viewrecord&from=export&id=L608829277 http://dx.doi.org/10.1371/journal.pone.0149082.

7 Kraemer WJ, Patton JF, Gordon SE, et al. Compatibility of high-intensity strength and endurance training on hormonal and skeletal muscle adaptations. *Journal of Applied Physiology* 1995;78(3):976–89.

8 Lundberg TR, Fernandez-Gonzalo R, Gustafsson T, et al. Aerobic exercise does not compromise muscle hypertrophy response to short-term resistance training. *Journal of Applied Physiology* 2013;114(1):81–89. doi:10.1152/japplphysiol.01013.2012 [published Online First: 25 October 2012].

9 McCARTHY JP, POZNIAK MA, AGRE JC. Neuromuscular adaptations to concurrent strength and endurance training. *Medicine & Science in Sports & Exercise* 2002;34(3):511–19.

10 Sale DG, MacDougall JD, Jacobs I, et al. Interaction between concurrent strength and endurance training. *Journal of Applied Physiology* 1990;68(1):260–70. http://www.embase.com/search/results?subaction=viewrecord&from=export&id=L20075241.

11 Spiliopoulou P, Zaras N, Methenitis S, et al. Effect of Concurrent Power Training and High-Intensity Interval Cycling on Muscle Morphology and Performance. *Journal of Strength and Conditioning Research* 2021;35(9):2464–71.

12 Terzis G, Spengos K, Methenitis S, et al. Early phase interference between low-intensity running and power training in moderately trained females. *European Journal of Applied Physiology* 2016;116(5):1063–73. doi:10.1007/s00421-016-3369-z [published Online First: 4 April 2016].

13 Tsitkanou S, Spengos K, Stasinaki A-N, et al. Effects of high-intensity interval cycling performed after resistance training on muscle strength and hypertrophy. *Scandinavian*

Journal of Medicine & Science in Sports 2017;27(11):1317–27. doi:10.1111/sms.12751 [published Online First: 23 September 2016].

14 Lundberg TR, Fernandez-Gonzalo R, Tesch PA. Exercise-induced AMPK activation does not interfere with muscle hypertrophy in response to resistance training in men. *J Appl Physiol (1985)* 2014;116(6):611–20. doi:10.1152/japplphysiol.01082.2013 [published Online First: 9 January 2014].

15 Nelson AG, Arnall DA, Loy SF, et al. Consequences of combining strength and endurance training regimens. *Physical Therapy* 1990;70(5):287–94.
